# Momentum quantization of hyperbolic phonon polaritons in photonic-crystal cavity

**DOI:** 10.1126/sciadv.aej0277

**Published:** 2026-10-28

**Authors:** Hwi Je Woo, Heejin Choi, Piyali Biswas, Seonyeong Kim, Hansung Kim, Sunae Seo, Suyong Jung, Eun Seong Lee, Junghoon Jahng, Chang-Won Lee

**Affiliations:** ^1^ Material Property Metrology Group, Korea Research Institute of Standards and Science (KRISS), Daejeon 34113, Republic of Korea.; ^2^ Institute of Advanced Optics and Photonics, Hanbat National University, Daejeon 34158, Republic of Korea.; ^3^ Department of Physics, Sejong University, Seoul 05006, Republic of Korea.; ^4^ Department of Applied Optics, School of Basic Sciences, Hanbat National University, Daejeon 34158, Republic of Korea.

## Abstract

Hyperbolic phonon polaritons (HPhPs) in van der Waals crystals enable extreme electromagnetic confinement and access to exceptionally large in-plane momenta, offering a powerful platform for deep-subwavelength nanophotonics. Although the lower Reststrahlen band (RS1) of hexagonal boron nitride (h-BN) is highly attractive for low-loss, strongly volume-confined polaritons, fully exploiting this potential remains challenging because RS1 modes are weakly accessible by conventional near-field detection and require efficient leakage suppression for high-Q confinement. Here, we demonstrate that momentum quantization of RS1 HPhPs enables high-Q, ultrasmall mode volume cavity states in an h-BN photonic-crystal nanocavity architecture. Periodic lateral cavity boundaries impose Fabry-Pérot phase matching, while the surrounding photonic-crystal lattice suppresses in-plane leakage and stabilizes discrete cavity-supported momentum states, transforming the continuous hyperbolic dispersion into a ladder of geometry-defined momentum eigenstates. Using photo-induced force microscopy, we directly visualize the quantized cavity modes in real space and reconstruct their discretized dispersion in momentum space. The resulting hyperbolic polaritonic cavity crystal exhibits quality factors exceeding 340 and ultrasmall mode volumes down to ${\bar{V}}_{m}=4.3\times 10^{-7}\;{\left(\lambda_{0}/n\right)}^{3}$, corresponding to Purcell enhancements approaching 10^7^. This approach establishes a route to high-Q, ultrasmall-volume RS1 polaritonic nanocavities with geometry-defined momentum states, providing a scalable platform for enhanced light-matter interactions, selective vibrational spectroscopy, and reconfigurable mid-infrared nanophotonic devices.

## INTRODUCTION

Hyperbolic phonon polaritons (HPhPs) in van der Waals (vdW) crystals provide a platform for nanophotonics, forming an intrinsically dense and continuous momentum space that enables dispersion engineering far beyond the diffraction limit. HPhPs support extremely large in-plane wavevectors ($k_{∥}\gg k_{0}$) with comparatively low dissipative loss, as their confinement arises from ionic lattice vibrations rather than free-carrier damping (*1*). Their strong optical anisotropy produces open isofrequency surfaces, enabling canalization (*2*–*4*), negative refraction (*5*, *6*), and enhanced *Q* factor/Purcell effect (*7*–*9*). Combined with the atomically thin and stackable nature of vdW crystals, which allows tunability via thickness, dielectric environment, and heterostructure design (*10*–*14*), HPhPs have established themselves as a leading platform for mid–infrared (IR) nanophotonics (*15*, *16*).

Among HPhPs, the lower Reststrahlen band (RS1) of hexagonal boron nitride (h-BN) is particularly attractive for low-loss and strongly volume-confined polaritonic states, offering a promising route toward high-*Q* resonances with ultrasmall mode volumes. However, fully exploiting this potential requires more than local-field confinement because polariton leakage and coupling to additional HPhP channels can limit the attainable *Q* factor. Previous approaches— including nanostructuring (*17*–*20*), waveguiding (*21*, *22*), and resonant confinement (*23*, *24*)—have demonstrated powerful control over HPhP propagation, localization, and resonances. Nevertheless, achieving high-Q RS1 HPhPs while in ultrasmall mode volumes remains challenging.

Here, we overcome this fundamental limitation by introducing a platform that enforces momentum quantization of HPhPs. By integrating an h-BN slab with a photonic-crystal (PhC) nanocavity architecture (Fig. 1A), forming a hyperbolic polaritonic-cavity crystal (HPCC), we transform the continuous hyperbolic dispersion into discrete set of momentum eigenstates. Periodic lateral boundaries impose Fabry-Pérot phase matching, while the PhC lattice acts as an effective momentum-selective cavity environment: The surrounding photonic bandgap suppresses in-plane radiative leakage (*25*, *26*), while distributed Bragg reflection reduces mode mixing and stabilizes discrete cavity-supported momentum states (*27*). This dual mechanism is fundamentally distinct from conventional cavity confinement: Rather than merely localizing fields, it selectively filters and stabilizes specific high-𝑘 momentum states, enabling high-*Q* polaritonic resonances within ultrasmall mode volume. The resulting HPCC establishes a direct mapping between geometry and momentum space. By tuning the cavity length through the PhC design, we achieve deterministic control over the allowed momentum states, effectively discretizing the high-𝑘 manifold into a ladder of addressable eigenstates.

**Fig. 1.**
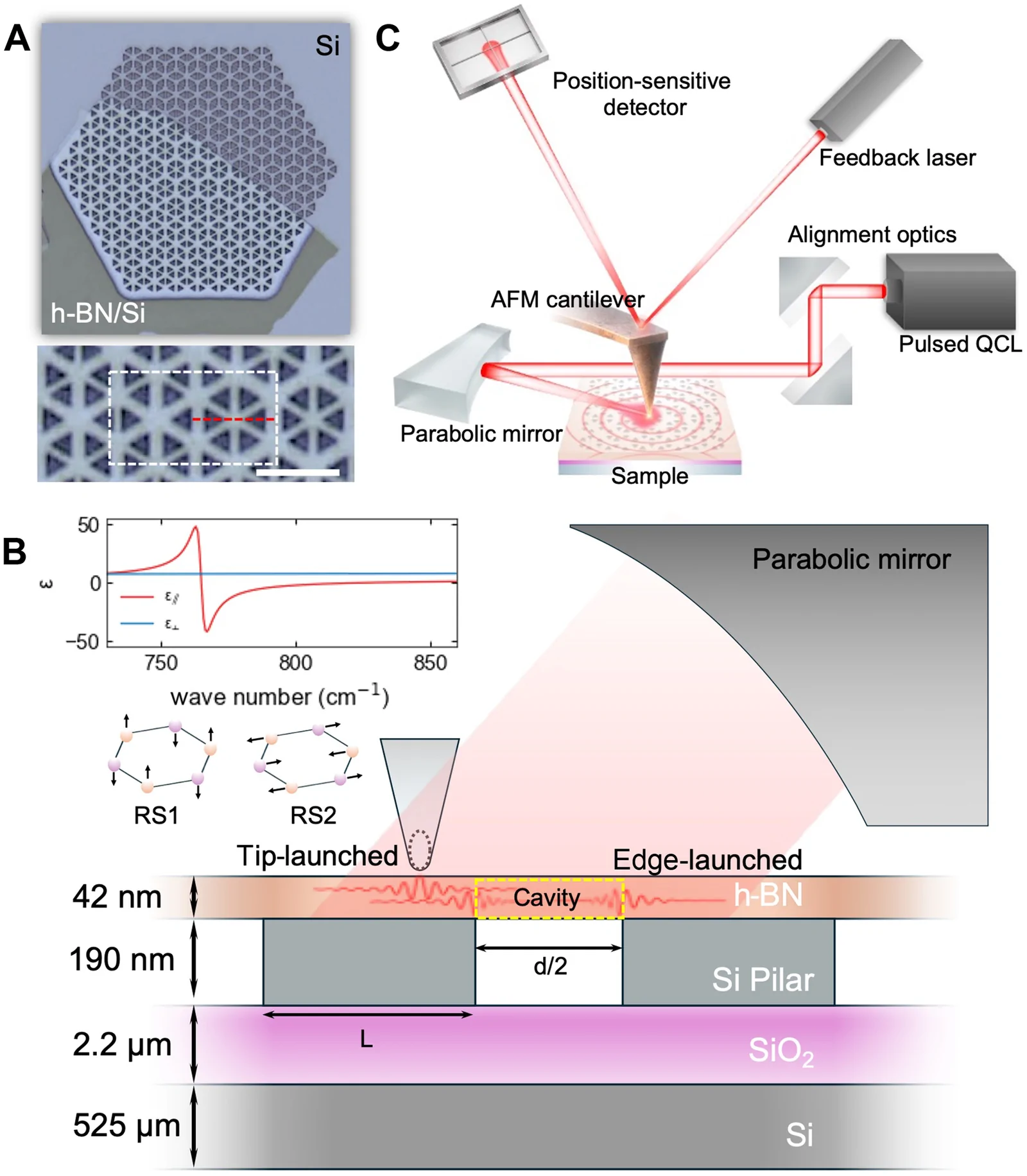
HPCCs based on h-BN in the RS1 band. (**A**) Schematic of HPCC structures and polaritons. The patterned Si is covered by a 42-nm h-BN slab, as shown in the OM image in (B). Scale bar, 5 μm. (**B**) Cross-sectional view of the HPCC showing the structural dimensions. The red waves represent polariton propagation, where type-I hyperbolicity (𝜀_⊥_ = 𝜀_𝑥_ = 𝜀_𝑦_ > 0, 𝜀_∥_ = 𝜀_𝑧_ < 0) occurs within the 765 to 824 cm^−1^ range of the RS1 band, as indicated by the calculated dielectric function of h-BN in the inset. The phonon vibration modes are illustrated in inset for RS1 (transverse mode) and RS2 (longitudinal mode). (**C**) Schematic of PiFM. Pulsed mid-IR radiation is focused onto the tip apex by a parabolic mirror. The tip- and edge-launched HPhPs form a standing-wave pattern in the sample, which induces local heating and thermal expansion. The resulting expansion deflects the AFM cantilever, and the cantilever motion is detected by a position-sensitive detector, thereby enabling mapping of the standing-wave pattern.

This momentum quantization is directly visualized using recent advanced nano–IR technique, photo-induced force microscopy (PiFM), which provides both real-space and spectroscopic access to the confined modes. Two-dimensional Fourier analysis reconstructs the discretized dispersion, while real-space imaging reveals a mode ordering of quantized cavity modes, including high-order HPhPs enabled by efficient edge launching and Fabry-Pérot phase matching. Quantitatively, the HPCC exhibits high *Q* factors (ω/Δω > 340) and ultrasmall mode volumes (${\bar{V}}_{m}=4.3\times 10^{-7}\;{\left(\lambda_{0}/n\right)}^{3}$), corresponding to Purcell enhancements approaching 10^7^. By using momentum quantization to stabilize high-*Q*, ultrasmall mode volumes of polaritonic states in cavities, our approach establishes a route to geometry-defined polaritonic nanocavities with direct real-space visualization and momentum-space indexing. This platform opens a scalable route toward enhanced light-matter interactions, selective vibrational spectroscopy, phonon-resonance control, and reconfigurable mid-IR photonic architectures.

## RESULTS

### A PhC platform for momentum-quantized HPhPs

h-BN is a paradigmatic platform for mid-IR HPhPs, supporting two Reststrahlen bands with opposite hyperbolic character (*28*, *29*). In the lower Reststrahlen band (RS1, ∼760 to 830 cm^−1^), h-BN exhibits type-I hyperbolicity ($\varepsilon_{⊥}>0,\varepsilon_{∥}<0$), where HPhPs are predominantly out of plane, volume-confined, and ultraslow (*v*_g_ ∼ c/500) (*29*, *30*). These properties make RS1 favorable for ultrasmall mode volumes and high-𝑄 cavity resonances. By contrast, the upper Reststrahlen band (RS2) supports type-II hyperbolicity dominated by in-plane phonon vibrations and more surface-localized polaritons. We therefore focus on RS1 as the optimal regime for realizing low-loss, cavity-confined high-𝑘 polaritonic states.

To impose lateral momentum quantization on polaritonic modes, we integrate an h-BN slab with a hexagonal PhC cavity with $C_{6v}$ symmetry formed by triangular Si pillars on SiO_2_ (Fig. 1B, fig. S1, and table S1). The pillars are displaced to form either shrunken or expanded cavity geometries. In this architecture, the laterally suspended h-BN span between two opposing Si-pillar sidewalls defines the HPCC, with characteristic half-lengths 𝑑/2 of ∼0.533 and 0.797 μm.

Under tunable mid-IR illumination, HPhPs propagate within the h-BN slab and undergo repeated reflection at the cavity boundaries, thereby forming deeply subwavelength standing-wave modes. These standing-wave modes constitute the laterally quantized RS1 cavity states targeted. To directly visualize these quantized modes in real space, we use a tip-based nano-optical imaging approach based on polariton interferometry (*25*, *31*). However, directly probing these RS1 modes is challenging for conventional scattering-type near-field optical microscopy (s-SNOM) (*28*) because the strongly volume-confined fields couple only weakly to the external near field (*29*), resulting in reduced scattering amplitude and poor standing-wave contrast.

To access this otherwise hidden regime, we use the PiFM technique (Fig. 1C), which transduces the highly confined internal field intensity into thermomechanical forces at the tip-sample junction (*32*). The resulting signal enables high-contrast mapping of the standing-wave intensity and, unlike s-SNOM, provides direct spectroscopic access to the cavity response without additional broadband sources or complex optical pathways. In this way, the HPCC platform makes it possible to directly visualize and spectroscopically resolve momentum-quantized, volume-confined HPhPs in the RS1 band.

As shown in the inset graph of Fig. 1B, the real part of the dielectric function (ε) of h-BN confirms type-I hyperbolicity over 765 to 824 cm^−1^, matching the spectral window used in this study. In this regime, HPhPs propagate as volume-confined nanorays (*33*, *34*), formed by the superposition of waveguided electric-field intensity ${|E|}^{2}$. The partial absorption of this sharply localized HPhP field locally heats the h-BN, and the amplitude of this heating follows the spatial distribution of the confined standing-wave intensity. Because the incident mid-IR beam focused onto the tip-sample junction is intensity-modulated at the sideband of tip resonance frequency, the local temperature rise and the resulting thermal expansion also oscillate at the same modulation frequency. This modulated expansion changes the tip-sample interaction force, generating a photo-induced thermal force (PiTF) that drives the cantilever at its mechanical resonance. By demodulating the cantilever motion, PiFM detects the local PiTF amplitude and therefore maps the confined HPhP standing-wave pattern with high contrast (note S1).

With this detection mechanism established, we turn to the direct observation of HPhP interference in the HPCC (Fig. 2A). The AFM tip-sample junction is illuminated by a focused, tunable mid-IR beam through a parabolic mirror, enabling simultaneous tip-launched and edge-launched excitation of HPhPs in the h-BN slab. The cavity sidewalls act as efficient scattering sites that launch propagating HPhPs, including higher-order modes (*35*), while also imposing hard lateral boundary conditions. The measured fringe pattern arises from interference between the phase-stable tip-launched HPhP field and the edge-launched field accumulated along the propagation path (*36*). The local field at the tip is therefore written as

Etot=Etip+Eedge(x) (1)

where, $E_{\text{tip}}=A_{\text{tip}}e^{i\phi_{\text{tip}}}$ and $E_{\text{edge}}=A_{\text{edge}}e^{i\left(k_{p}x+\phi_{\text{edge},0}\right)}$. ϕ and $k_{p}$ denote the phase and the wave number of polaritons, respectively. The corresponding PiFM signal is proportional to the local field intensity, I(x)

$$I\left(x\right)\propto {|E_{\text{tip}}+E_{\text{edge}}\left(x\right)|}^{2}$$ (2)

which yields

$$I\left(x\right)\propto A_{\text{tip}}^{2}+A_{\text{edge}}^{2}+2A_{\text{tip}}A_{\text{edge}}\text{cos}\left(k_{p}x+\phi_{\text{sample}}-\phi_{\text{tip}}\right)$$ (3)

**Fig. 2.**
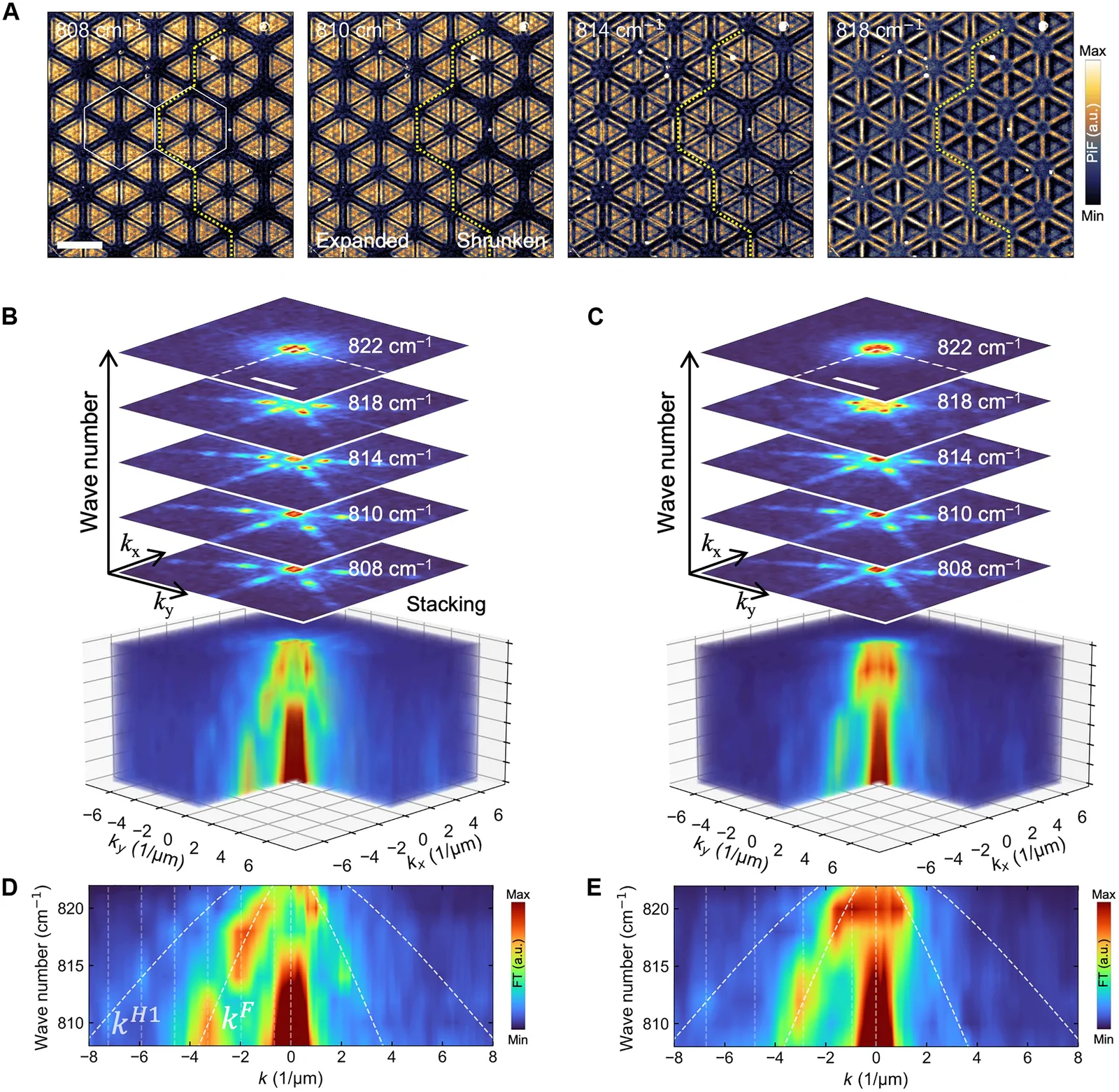
PiFM results of multi-unit cells. (**A**) PiFM images for multi-uni10t cells. The left part consists of expanded HPPCs, and the right part consists of shrunken HPCCs. Scale bar, 3 μm. (**B** and **C**) FT images vertically stacked along the frequency axis for expanded and shrunken HPCCs, respectively. The dispersion relations of HPhPs are reconstructed from PiFM measurements. (**D** and **E**) Plane views of the HPhP dispersion relations along *x* (negative value) and *y* (positive value) axis. The white dashed curves indicate HPhPs modes calculated analytically (eq. S7). $k^{F}$ and $k^{H1}$ represent the fundamental and first high-order HPhP modes. The vertical white dashed lines represent quantized modes calculated by Eq. 5.

Thus, the spatial oscillation period in the PiFM image directly encodes the HPhP momentum $k_{p}$. This phase-referenced interference mechanism accounts for the periodic contrast observed in Fig. 2A, where PiFM images acquired at selected quantum cascade laser (QCL) wave numbers resolve HPhP fringes with deep-subwavelength resolution ($∼\lambda_{0}/500$). The interference patterns differ between the expanded and shrunken HPCCs, motivating the momentum-space analysis presented below.

### Direct observation of discrete momentum eigenstates

To directly resolve the momentum states supported by the HPCC, we stack the Fourier-transform (FT) images of PiFM data along the frequency axis to reconstruct three-dimensional dispersion volumes (Fig. 2, B and C). Slicing these FT cubes along both $k_{x}$ and $k_{y}$ yields momentum-frequency cross sections that reveal the cavity-selected momentum states in the expanded and shrunken geometries. The extracted FT maps (Fig. 2, D and E) display the defining signatures of cavity phase matching, including both the fundamental ($k^{F}$) and higher-order ($k^{H1}$) HPhP branches. The vertical dashed lines mark the Fabry-Pérot phase-matching conditions imposed by the lateral cavity length 𝑑/2

$$\left(2n+\frac{1}{2}\right)\cdot \lambda_{p}=\frac{d}{2}\;\text{for even mode}$$ (4)

$$\left[\left(2n+1\right)+\frac{1}{2}\right]\cdot \lambda_{p}=\frac{d}{2}\;\text{for}\;\text{odd}\;\text{mode}$$ (5)

or equivalently,

$$\left(2n+\frac{1}{2}\right)\cdot \frac{2}{d}=k_{p}\;\text{for even mode}$$ (6)

$$\left[\left(2n+1\right)+\frac{1}{2}\right]\cdot \frac{2}{d}=k_{p}\;\text{for}\;\text{odd}\;\text{mode}$$ (7)

Here, *n* is the integer quantization index. These expressions show explicitly that the allowed in-plane momentum scales inversely with the cavity dimension. When the continuous HPhP dispersion intersects these cavity-imposed phase-matching conditions, discrete resonances emerge. These quantized states are absent in h-BN on planar Si (figs. S4, table S2, and note S3), demonstrating that the discrete branches arise from the HPCC boundary conditions rather than from the intrinsic dispersion of h-BN itself. Quantization is observed only along $k_{x}$, consistent with the fact that the lateral cavity is defined solely by two opposing Si-pillar sidewalls with $C_{6v}$ symmetry.

To isolate the physical origin of this quantization, we performed full-wave simulations comparing two canonical structures: a single-pillar geometry and a multimodal cavity formed by two neighboring Si pillars (Fig. 3, A and B, and note S4). Real-space fringe positions alone do not identify the polariton momentum. Therefore, we determine the momentum states from two-dimensional Fourier analysis and direction-resolved k-space cross sections. This comparison cleanly separates the role of a single dielectric boundary from that of a finite lateral cavity. In the single-pillar geometry, the Si edge efficiently launches HPhPs into both supported and suspended regions, but the resulting interference follows the continuous dispersion expected for planar waveguided or nanoray modes, with no evidence of quantization (Fig. 3, C and E). In sharp contrast, the multimodal cavity produces strong standing-wave fringes in the suspended region and discrete momentum branches that satisfy the Fabry-Pérot conditions of Eqs. 4 to 7 (Fig. 3, D and F). Notably, higher-order branches are absent in the single-pillar structure, establishing that a finite cavity—not a single scattering boundary—is required to convert the continuous HPhP manifold into a discrete ladder of quantized momentum states.

**Fig. 3.**
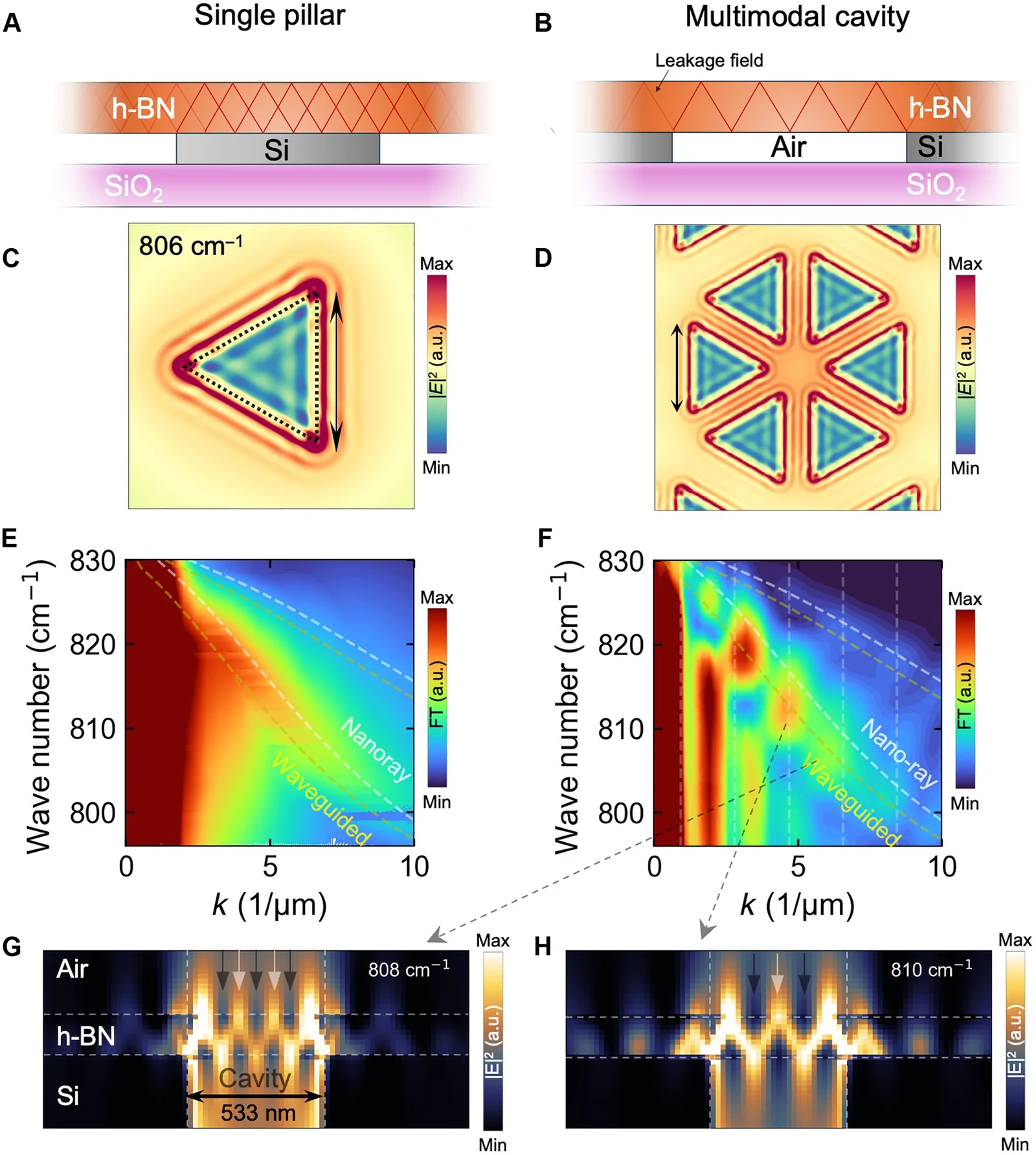
FDTD simulation for canonical h-BN slab configurations. (**A** and **B**) Schematics of single pillar and multimodal cavity geometries. The red lines represent zig-zag motion of HPhP nanorays in the h-BN slab. (**C** and **D**) FDTD simulated |*E*|^2^ field distributions in *xy* plane. The length of edges for triangles is 1.364 μm (black arrows). (**E** and **F**) Fourier transformed images for each |*E*|^2^ field simulation. The yellow and white dashed curves given by eqs. S7 and S8, respectively. (**G** and **H**) Cross-sectional |*E*|^2^ field simulations of the multimodal cavity. Arrows denote each corresponding mode in (F).

This interpretation is further supported by simulated cross-sectional |*E*|^2^ maps (Fig. 3, G and H, and fig. S6A), which reveal node and antinode sequences characteristic of Fabry-Pérot–like cavity quantization. The suspended region supports confined nanorays, while a leakage field extends into the supported regions. As the excitation wave number increases from 808 to 810 cm^−1^, the polariton wavelength increases and the number of antinodes and nodes accommodated within the fixed cavity decreases (antinodes: 2 → 1 and nodes: 3 → 2), directly visualizing the transition between adjacent quantized momentum states. By contrast, the inverse geometry maintains a continuous waveguided response without discrete, wave number–selective node counting (fig. S6B), confirming that hard lateral cavity boundaries are essential for momentum quantization. Thus, the emergence of well-defined quantized and higher-order branches in the two-pillar geometry is consistent with the multimodal reflection, where boundary reflection and higher-order HPhP components help suppress leakage and form confined cavity states (*7*).

### Real-space imaging of geometry-programmed momentum states

To verify how these quantized momentum states manifest at the scale of a single cavity, we image one HPCC unit cell at high magnification with PiFM and FT the same field of view (Fig. 4, A and B). PiFM resolves the cavity mode profiles with a spatial resolution of ∼23 nm. The top panels show standing-wave fringes both on the pillar surfaces and in the suspended cavity region, while the bottom panels display the corresponding two-dimensional momentum-space maps. These measurements directly track geometry-programmed HPhP momenta in real and reciprocal space.

**Fig. 4.**
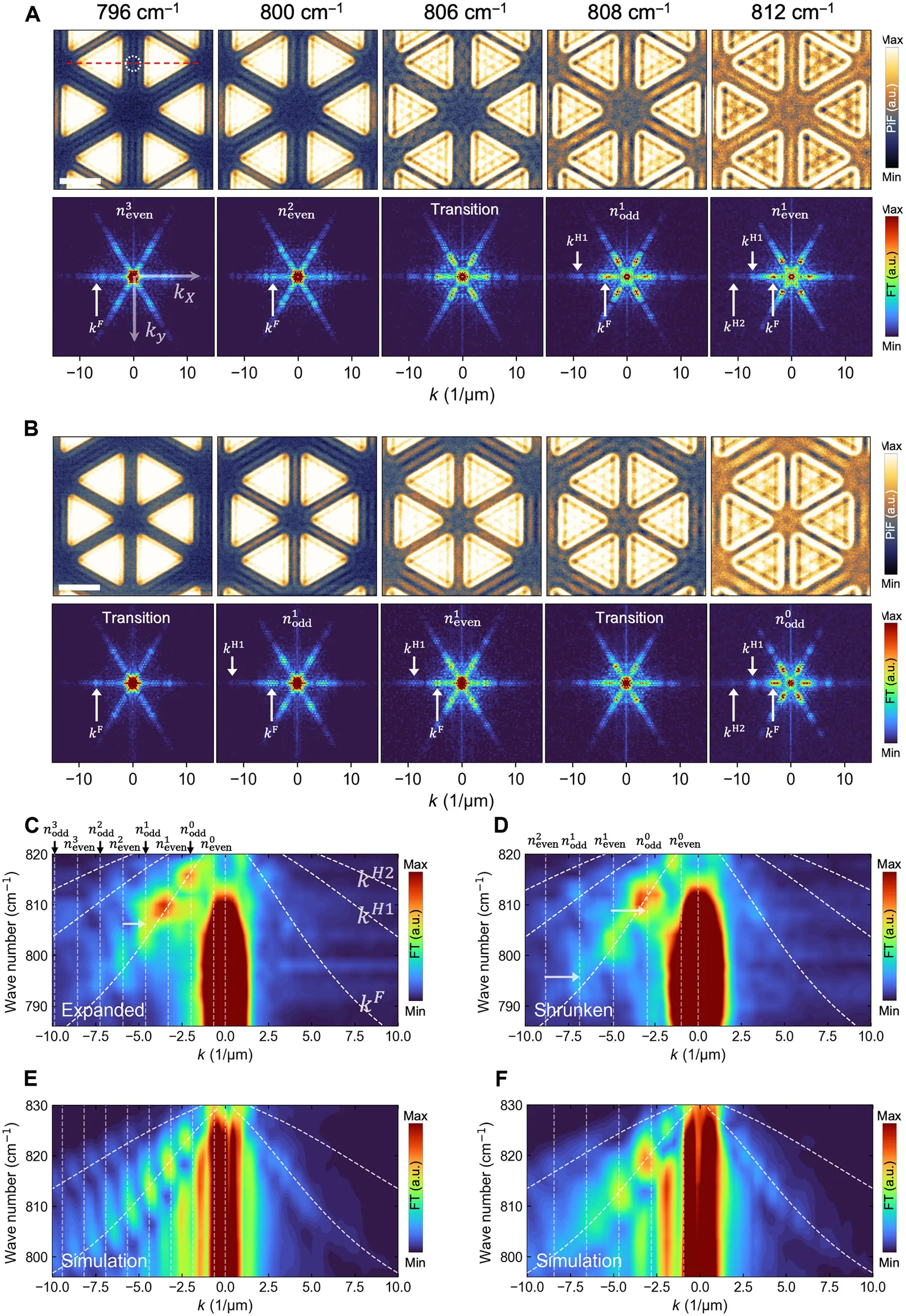
High-magnification PiFM images in HPCCS. (**A** and **B**) Top panels: Spectral PiFM images for a single unit cell. Scale bar, 1 μm. Bottom panels: corresponding Fourier transformed images. (**C** and **D**) Stacked FT images along frequency. White dashed curves are calculated dispersion of suspended waveguide modes of HPhPs. The white arrows indicate momentum transitions. (**E** and **F**) FDTD simulation results for stacked FT images along frequency.

A frequency series reveals the evolution of quantized-state transitions in the expanded and shrunken HPCCs. At 796 cm^−1^, the expanded HPCC exhibits three well-defined fringes near the cavity gap, whereas the fringes in the shrunken HPCC are indistinct, consistent with the transition from $n_{\text{even}}^{2}$ to $n_{\text{odd}}^{1}$ (Fig. 4, C and D). At 800 cm^−1^, the expanded cavity supports two fringes, while the shrunken cavity shows a single fringe. At 806 cm^−1^, the fringes in the expanded geometry become indistinct as the mode approaches the next quantized transition. At 808 cm^−1^, the trend reverses: The shrunken HPCC approaches $n_{\text{even}}^{1}\to n_{\text{odd}}^{0}$, whereas the expanded cavity still sustains a single fringe. At 812 cm^−1^, the shrunken HPCC reaches the $n_{\text{odd}}^{0}$ state, while the expanded cavity retains a single confined fringe. In reciprocal space, the corresponding FT peaks split into multiple higher-order components (white arrows), directly reflecting the emergence of discrete cavity-selected momentum states.

Following the same FT analysis used in Figs. 2 and 4 (C and D) plot the measured momentum-frequency dispersion along $k_{x}$ and $k_{y}$, together with the calculated suspended-h-BN dispersion (white dashed curves). Figure 4 (E and F) shows the corresponding finite-difference time-domain (FDTD) simulations for the expanded and shrunken geometries. For both structures, the measured branches appear at substantially larger in-plane momentum than in planar h-BN/Si (fig. S4), and the fundamental branch is accompanied by resolved higher-order branches ($k^{H1}$ and $k^{H2}$; fig. S8). Together, these results show that the HPCC in the RS1 band imposes hard lateral boundary conditions that quantize the in-plane momentum into a discrete ladder of cavity-allowed states. When edge-launched polariton waves satisfy the Fabry-Pérot phase-matching condition, leakage is suppressed and propagation is sufficiently long-ranged to generate high-𝑄 resonances. The quantized eigenstates therefore appear simultaneously as well-separated branches in Fourier space and as distinct real-space fringe patterns.

### High-*Q* and ultrasmall-volume quantized cavity states

Having established the real-space and momentum-space signatures of quantization, we next examine the spectral response of the HPCC cavities. Experimental PiFM spectral cross sections extracted along the red dashed line in Fig. 4A are shown in Fig. 5, A and B for the expanded and shrunken HPCCs, respectively. In these local cavity spectra, the confined nanorays appear as discrete resonances that can be indexed by the quantization number 𝑛 defined in Eqs. 6 and 7. Both the lowest-order and higher-order modes are resolved, demonstrating that the phase-matched HPhP branches evolve into spectrally discrete standing-wave cavity modes under lateral confinement.

**Fig. 5.**
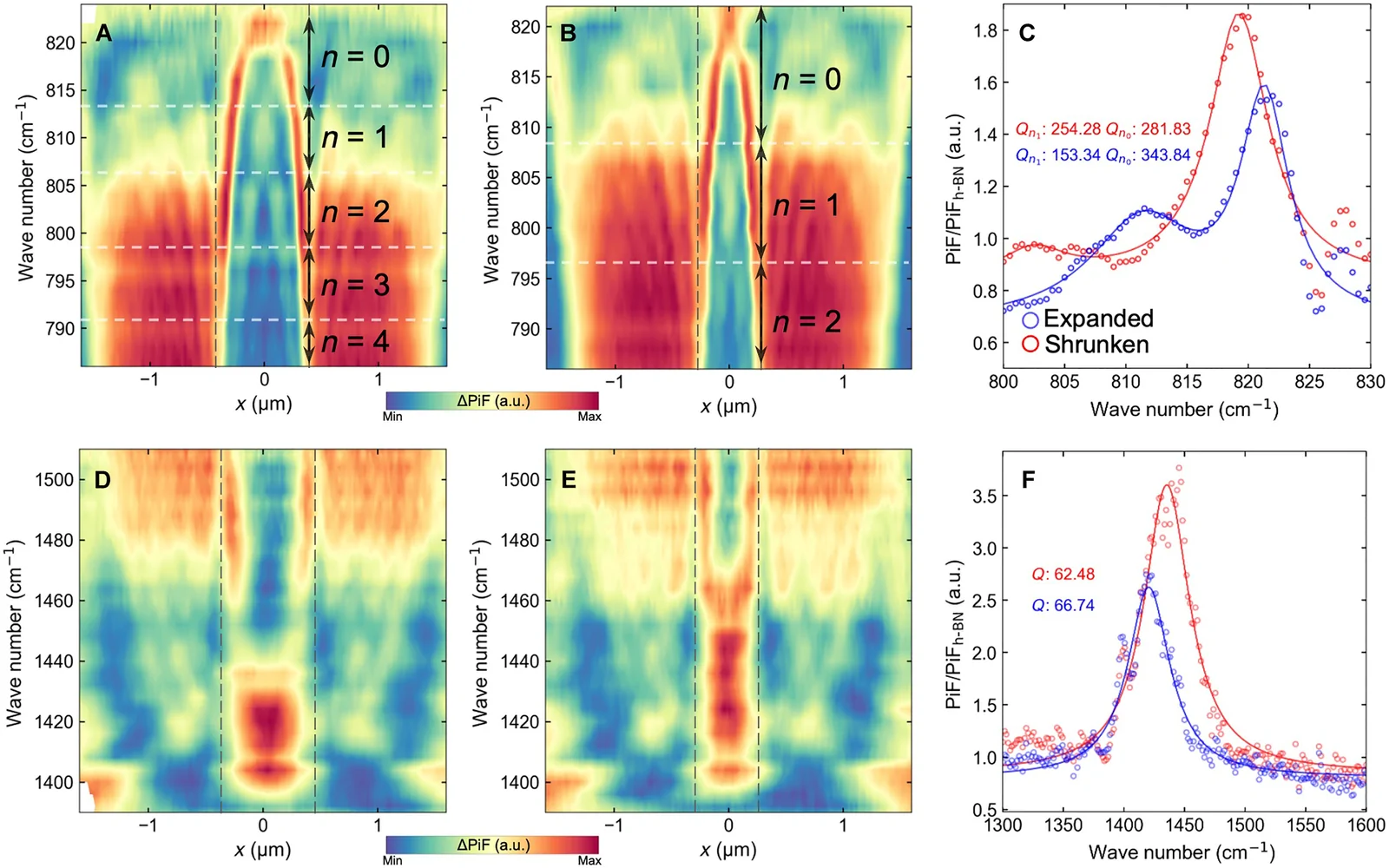
Quantized HPhPs in HPCC cavities and *Q* factors of HPCCs. (**A** and **B**) Spectral line profiles of HPCCs in the RS1 band [(A) expanded, (B) shrunken], extracted along the red dashed line in Fig. 4A. (**C**) PiFM spectra in the RS1 band measured at the white dashed circle in Fig. 4A (blue circles: expanded HPCC, red circles: shrunken HPCC, blue and red curves are fitted results with multiple gaussians). (**D** and **E**) Spectral line profiles of HPCCs in the RS2 band. (**F**) PiFM spectra in the RS2 band.

For the expanded HPCC, the lowest index (*n* = 0) appears above 814 cm^−1^, followed sequentially by *n* = 1 at 814 to 806 cm^−1^, *n* = 2 at 806 to 798 cm^−1^, *n* = 3 at 798 to 792 cm^−1^, and *n* = 4 below 792 cm^−1^. For the shrunken HPCC, *n* = 0 appears above 808 cm^−1^, followed by *n* = 1 in 808 to 796 cm^−1^, and *n* = 2 below 796 cm^−1^. These assignments agree closely with the FDTD simulations (fig. S9A), confirming the deterministic relation between cavity geometry and the accessible momentum ladder.

The spectral sharpness of these modes is summarized in Fig. 5C. PiFM spectra measured at the cavity center (white circle in Fig. 4A) show pronounced peaks associated with the quantized cavity indices. Multi-Lorentzian fitting yields quality factors $Q_{n_{0}}$ exceeding 340 and 280 for the lowest-order modes in the expanded and shrunken HPCCs, respectively (821.3 and 819.2 cm^−1^). For *n* = 1, the corresponding quality factors, $Q_{n_{1}}$, are 153.3 and 254.2, with the reduced value in the expanded lattice reflecting greater propagation loss over the longer cavity path. These values establish that momentum-quantized RS1 HPhPs can simultaneously support discrete mode indexing and exceptionally high spectral purity.

This behavior is unique to RS1. In the RS2 band, by contrast, the cavities exhibit only weak resonances without clear momentum quantization more than 1360 to 1480 cm^−1^ (Fig. 5, D and E). Although spectral features are still observed, they are much broader (Q_cav_ < 70) and do not evolve into a discrete, indexable momentum ladder (Fig. 5F). This contrast highlights the essential role of type-I, volume-confined, low-loss HPhPs in enabling robust momentum quantization. The higher phonon loss, faster group velocity, and more surface-like character of RS2 polaritons inhibit higher-order confinement and suppress the emergence of well-resolved cavity eigenstates (*28*, *29*).

Last, the HPCC platform combines these high quality factors with extreme spatial confinement. In the RS1 band, the shrunken HPCC reaches a mode volume as small as ${\bar{V}}_{m}=4.3\times 10^{-7}\;{\left(\lambda_{0}/n\right)}^{3}$ (note S5), corresponding to confinement several orders of magnitude tighter than in conventional dielectric cavities. When combined with the measured high-𝑄 resonances, this yields an extraordinary Purcell enhancement of $F=4.93\times 10^{7}$ (eq. S10). The simultaneous realization of discrete momentum quantization, high-𝑄 spectral selectivity, and ultrasmall mode volume establishes the HPCC as an unprecedented dielectric platform for wave vector–engineered light-matter interactions at the nanoscale.

## DISCUSSION

The ability to quantize polariton momentum provides a route to overcoming a central limitation of hyperbolic polaritons: realizing high-*Q* and ultrasmall-volume RS1 HPhP cavity states in a geometry-defined manner. In extended hyperbolic media, the accessible high-*k* polariton response is not constrained by finite cavity phase-matching conditions, so polariton energy can couple to multiple propagation channels and leak away rather than forming long-lived resonant states. This makes it difficult to reach high-*Q* factors while preserving the ultrasmall mode volumes enabled by RS1 volume confinement. By contrast, cavity-induced momentum quantization selects discrete phase-matched wave vector states, enabling polaritons to be stabilized as high-*Q* resonances while maintaining ultrasmall mode volumes. This discrete momentum basis establishes a direct correspondence between cavity geometry and the supported polaritonic states, thereby linking the cavity dimensions to the allowed momentum states, resonance order, quality factor, and mode volume. More broadly, it provides a foundation for high-*Q*, ultrasmall-volume polaritonic nanocavities and enhanced light-matter interactions in deeply subwavelength systems.

## MATERIALS AND METHODS

### HPCC fabrication

The HPCCs were fabricated on silicon-on-insulator wafers consisting of a 190-nm-thick top silicon device layer and a 2.2-μm-thick buried oxide layer. A negative electron-beam resist (hydrogen silsesquioxane) was spin-coated onto the wafer, followed by patterning using a 100-kV electron-beam lithography system. After development, the exposed silicon was etched using a standard HBr-based dry etching process in an inductively coupled plasma reactive ion etcher. The remaining resist mask was removed by HF solution. To eliminate any residual contaminants, the sample was cleaned in a Piranha solution. Subsequently, the h-BN flake with a thickness of about 42 nm was transferred onto the photonic crystal using a polydimethylsiloxane-based dry transfer method.

### Near-field measurement

PiFM was used to image HPhPs with deep-subwavelength resolution. Measurements were performed on a commercial AFM platform (Vista One, Molecular Vista Inc.) equipped with a mid-IR QCL tunable across the RS1 and RS2 bands (751 to 1900 cm^−1^). A PtIr-coated cantilever (PPP-NCLPt, Nanosensors) was operated in noncontact mode, with its second eigenfrequency near 1 MHz and oscillation amplitude kept below 1 nm. The QCL beam, modulated at the sum of the first and second cantilever resonances ($f_{1}+f_{2}$), was focused onto the tip-sample junction using a parabolic mirror. The PiFM signal was demodulated at $f_{1}$ (∼150 kHz) to isolate the tip-enhanced thermal force channel, ensuring high sensitivity to volume-confined RS1 modes. Polariton fringes were recorded by raster scanning the sample over the HPCC region while synchronously acquiring topography.

### Near-field simulation

Three-dimensional FDTD simulations (Ansys Lumerical) were used to calculate the near-field ∣E∣2 distribution in a realistic HPCC model, reproducing the experimental geometry and excited in the RS1 band with PML boundaries. More details are presented in Supplementary Note S4.

## Supplementary Materials

### This PDF file includes:

Figs. S1 to S11 Tables S1 and S2 Supplementary Notes S1 to S6 References
