# Supplementary material for: Momentum quantization of hyperbolic phonon polaritons in photonic-crystal cavity
Source: Sci Adv. 2026 Oct 28;12(44):eaej0277. doi: 10.1126/sciadv.aej0277 (PMC13715691; doi:10.1126/sciadv.aej0277)
Supplement: Supplementary file 1 [file sciadv.aej0277_sm.pdf]

Science Advances
AAAS

Supplementary Materials for

Momentum quantization of hyperbolic phonon polaritons in photonic-crystal cavity

Hwi Je Woo *et al.*

Corresponding author: Eun Seong Lee, eslee@kriss.re.kr; Junghoon Jahng, phyjjh@kriss.re.kr; Chang-Won Lee, cwlee42@hanbat.ac.kr

Sci. Adv. **12**, eaej0277 (2026)
DOI: 10.1126/sciadv.aej0277

This PDF file includes:

Figs. S1 to S11
Tables S1 and S2
Supplementary Notes S1 to S6
References

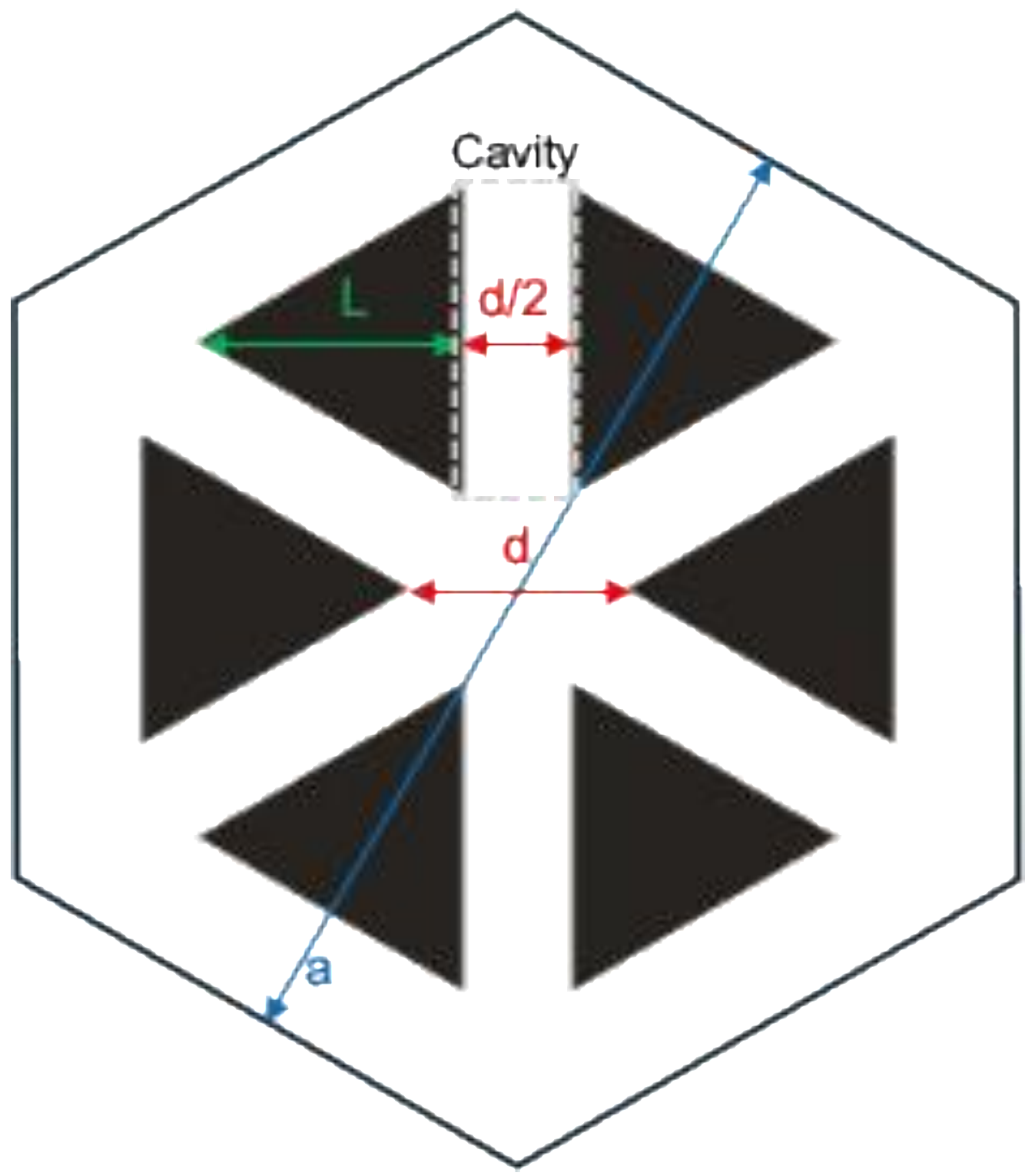

Fig. S1. Parameters of HPCCs.

Parameters	Target size (μm)	Estimated parameters in AFM (μm)	Error (%)
a	4.4	-	-
L	1.181	-	-
d/2 (shrunken)	0.533	0.52	2.5
d/2 (expanded)	0.797	0.76	4.2

Table S1. Parameters of HPCCs.

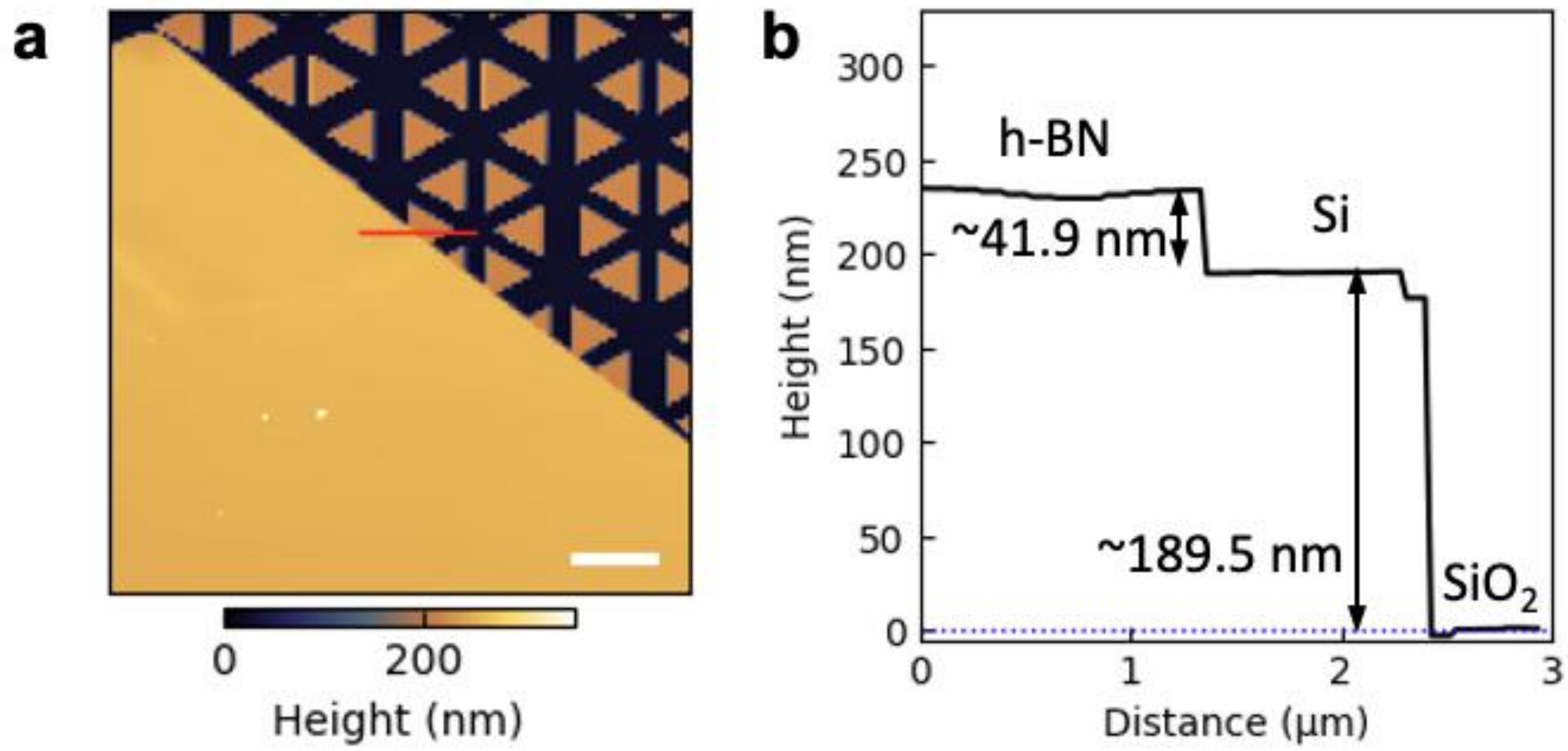

Fig. S2. AFM image of bare PhC and thickness of h-BN. (a) Topographic image of the h-BN/PhC. The scalebar represents 5 μm. (b) Corresponding a height profile measured along the red line in (a)

Supplementary Note 1. Detection of photo-induced thermal force(*32*, *37*, *38*)

In PiFM measurements, two distinct light–matter force channels can drive the cantilever response: photo-induced dipole forces (PiDF) and photo-induced thermal forces (PiTF). PiDF arise from optically induced dipoles at the tip and sample that interact via Coulomb forces; this mechanism underpins the signal contrast in conventional s-SNOM. In highly polarizable media (metals, plasmonic substrates), PiDF can reach tens of piconewtons, whereas in weakly polarizable dielectrics (e.g., organic polymers) they are typically one–two orders of magnitude smaller.

PiTF, by contrast, originate from absorptive heating and the ensuing thermal expansion of the sample. Under resonant illumination (*e.g.*, near molecular/phonon vibrational bands), optical absorption produces a thermal expansion within the near-field interaction volume; the resulting total thermal expansion can be defined as:

$$\Delta L_{tot}(t) = \frac{\sigma l_z \tau_{th}}{\rho C V_{heat}} \int \alpha_{abs} \frac{1}{2} c\varepsilon_0 \left(|E_f + E_n(z')|^2\right) dV_{abs} \tag{S1}$$

where σ, l_z, ρ, C, and α_{abs} are the linear thermal expansion coefficient, heating length, density, heat capacity, and absorption coefficient of the sample. z', c, E_f, and E_n are the sample thickness, speed of light, vacuum permittivity, far- and near- fields intensities. τ_{th} is the effective thermalization time (considering proper heating and cooling time). With the light modulated as sideband (f_m = $f_1 + f_2$, f_i: i^{th} resonant frequency of cantilever), the total thermal expansion $\Delta L_{tot}(t) = \Delta L_g(t) + \Delta L_t(t)$ can be divided as:

$$\Delta L_g(t) \approx \Delta L_g \sin(f_m t + \theta_m)\,, \tag{S2}$$

$$\Delta L_t(t) \approx \Delta L_t(z_0) \sin(f_m t + \theta_m) + \frac{\partial \Delta L_t}{\partial z} I_{z_0} A_2 \sin(f_2 t + \theta_s) + O(\epsilon)\,, \tag{S3}$$

where, ΔL_g is global thermal expansion from far-field absorption, and ΔL_t is tip-enhanced thermal expansion from near-field absorption. A_2 is the amplitude of cantilever's driving. In the non-contact (attractive) region, the measured force which induced by thermal expansion where the tip-sample distance modulated by external modulated light, can be estimated as Derjaguin-Muller-Toporov (DMT) model. Then, we can obtain the forces as:

$$F_{th}^{g} \approx -\frac{2\pi H_{eff} R \Delta L_g}{3} \left[\frac{Z_g}{Z_g^2 - R^2}\right], \tag{S4}$$

$$F_{th}^{t} \approx -\frac{2\pi H_{eff} R \Delta L_t}{3} \left[\frac{(\delta R Z_t)^2}{(Z_t^2 - R^2)(Z_t^2 - R^2 + \delta^2 R^2)}\right], \tag{S5}$$

where $Z_j = z + R + \Delta L_j$ with $j = g$ or t and R is the radius of the tip apex. **Eq. S5** highlights the strong distance sensitivity (z^{-3}) of the tip-enhanced thermal force than global thermal expansion force (z^{-1}).

In practice, PiFM leverages this pathway selectivity by modulating the illumination and demodulating at a cantilever resonance (sideband scheme), which preferentially isolates the tip-enhanced thermal expansion force signal with high sensitivity. The resulting periodic thermal expansion generates a PiTF beneath the AFM tip, producing corresponding surface displacements that are detected by the cantilever, as illustrated in **Fig. S3a**. By modulating the incident mid-IR beam (at sideband $f_m = f_1 + f_2$), and demodulating the cantilever response at the first resonant frequency (f_1) of the tip, sideband PiFM selectively detects this PiTF signal(*2*, *3*), yielding high-contrast fringe maps that directly visualize the underlying HPhP interference pattern.

Because the PiTF contrast originates from thermally induced surface displacements rather than direct optical scattering, it is essential to confirm that the observed fringes are not artifacts of the surface morphology. **Figs. S3b** and **S3c** present the AFM topography and corresponding PiFM images of the HPCC region: The AFM shows wrinkles and small bubbles in the h-BN layer but does not resolve the underlying Si-pillar lattice, indicating that the height signal is dominated by h-BN surface irregularities rather than the periodic cavity structure. In contrast, PiFM images clearly resolved underlying features. This distinction demonstrates that the PiFM signals cannot arise from static topographic modulation. Instead, they originate from the engineered near-field distribution within the HPCC, where HPhP standing waves generate spatially periodic electric field, producing the thermal expansion forces that PiFM measures with high fidelity. Throughout this work we therefore focus on tip-enhanced thermal expansion and the resulting PiTF contribution, treating any PiDF and global thermal expansion force background as negligible under our experimental conditions.

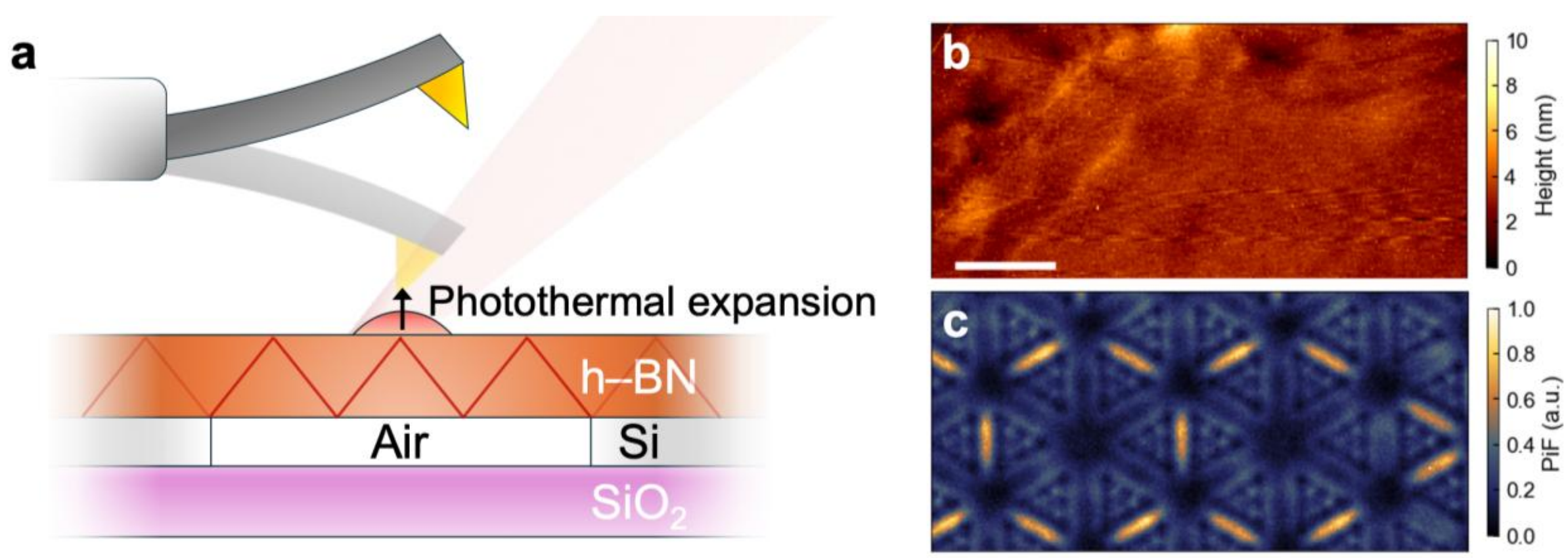

Fig. S3. Photo-induced thermal expansion of PiFM (a) The periodic waves induce PiTF under the AFM tip. (b, c) Demonstration that topographic artifacts do not affect PiFM measurements. The topographic image (b) and PiFM image (c) are obtained simultaneously. The scale bar represents 2 μm. The graphic was created by the authors using Microsoft PowerPoint.

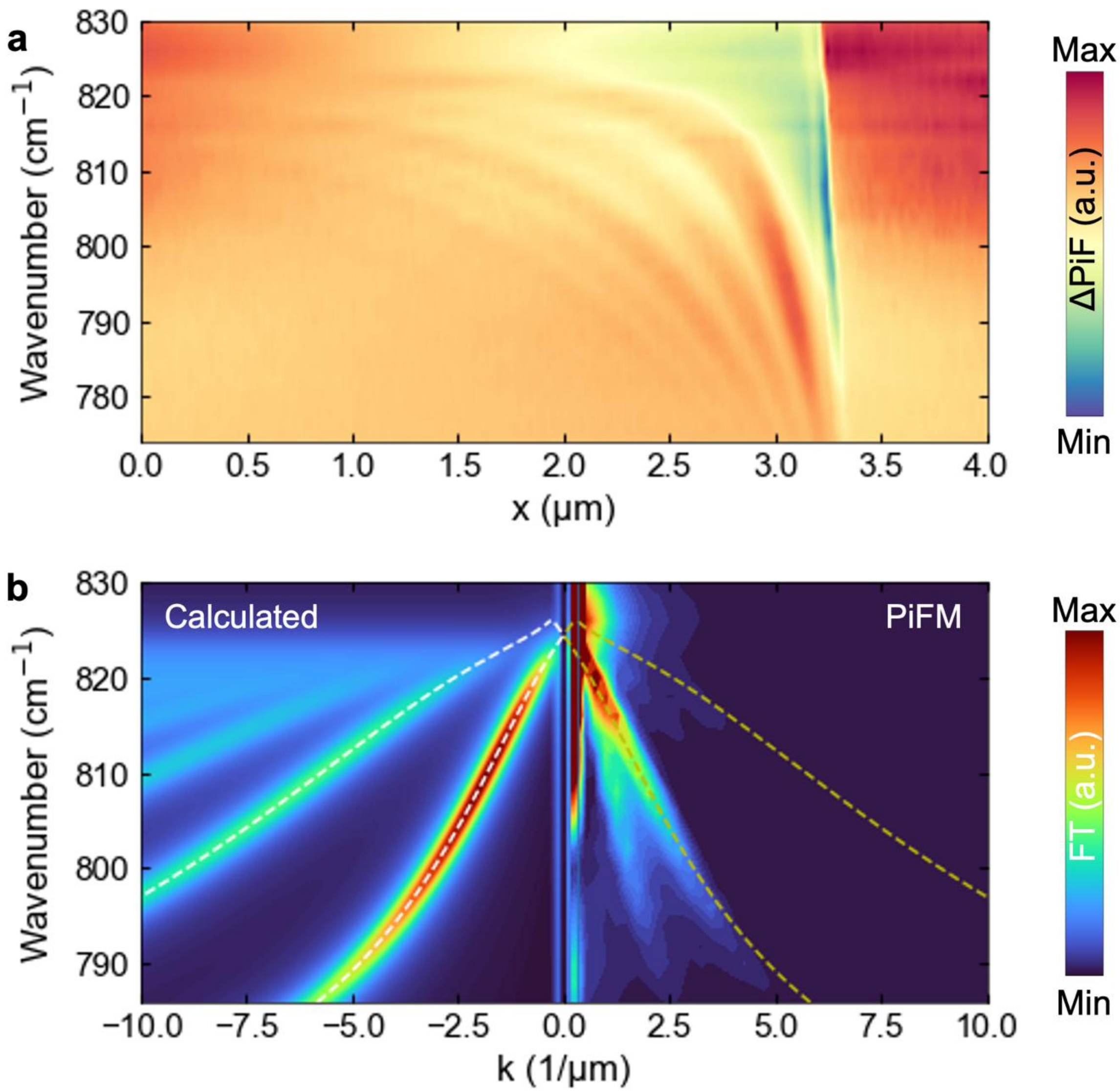

Fig. S4. HPhP of h-BN/planar Si substrate. (a) Spectral line profiles of h-BN on planar Si substrate. (b) Calculated (left, see **Supplementary Note 3**) and experimentally obtained dispersions by applying Fourier transform on (a). The yellow dashed line represents calculated dispersion following **Eq. S7**.

unit: cm^{-1}	ε_{inf}	ω_{TO}	ω_{LO}	Γ
RS1	2.95	765	824	4
RS2	4.87	1375	1610	5

Table S2. Parameters for h-BN phonon modes.

Supplementary Note 2. Numerical interference model

To test whether the discrete features in our dispersion maps could arise solely from surface interference on the Si-supported regions, we constructed a minimal ‘supported-only’ model: polaritonic cosine waves were launched from the edges of each triangular Si pillar, fields were evaluated only on the Si-supported surface, and propagation through the suspended cavity was explicitly excluded (**Fig. S5a**; top: real-space amplitude, bottom: 2D FT). We then repeated the **Figs. 2** and **4** workflows—stacking FT maps while sweeping geometry—first varying the cavity length **d** at fixed pillar-edge length **L**, then varying **L** at fixed **d** (**Fig. S5b**; top: **d**-sweep, bottom: **L**-sweep).

As expected for standing waves confined to the supported surface, discrete FT maxima do appear; however, they are insensitive to **d** and shift only with **L**. In other words, a surface-only interference picture cannot imprint the **d**-dependence that characterizes cavity quantization in the main text. By contrast, the experiment (**Figs. 2** and **4**) shows that the shrunken and expanded HPCCs—designed with the same **L** but different **d**—exhibit different sets of discrete modes.

In the RS1 regime, where HPhPs are bulk-like and ultraslow, the suspended cavity participates in the propagation–reflection loop and enforces the Fabry–Pérot quantization condition **Eq. 5**. The observed discreteness and its selective sensitivity to **d** therefore identify cavity quantization—not surface standing waves along the Si pillars—as the origin of the dispersion and mode ordering seen in **Figs. 2d** and **2e**.

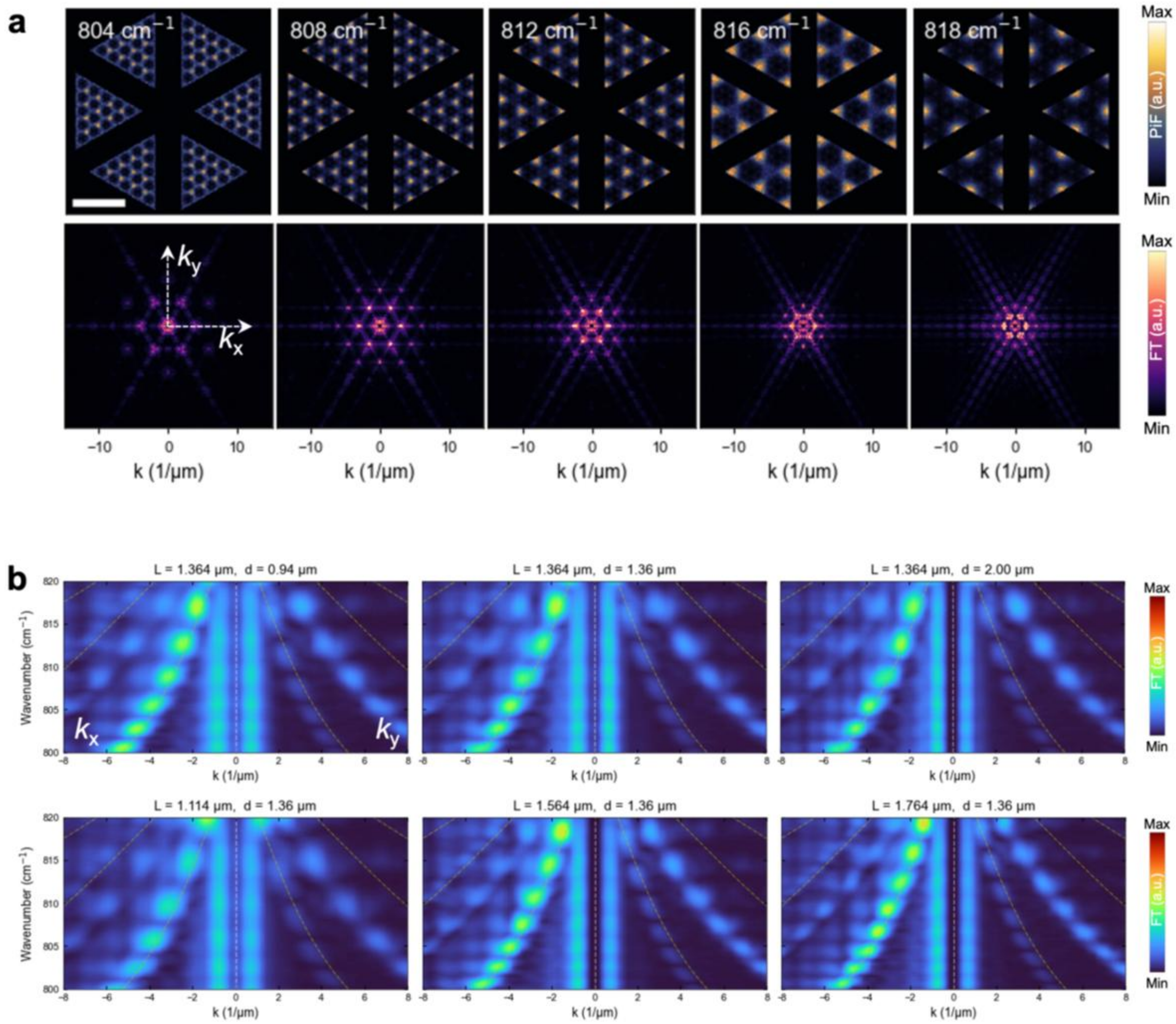

Fig. S5. Numerical simulations of HPhPs in HPCCs under a supported-only model. (a) shows the real-space patterns (top) and their two-dimensional Fourier transforms (bottom). (b) FT results of numerically simulated HPCCs (top row: **d** sweep and bottom row: **L** sweep).

Supplementary Note 3. Dispersion of hyperbolic phonon polariton.

To derive dielectric function of h-BN, Lorentz-Drude model is introduced as:

$$\varepsilon = \varepsilon_{\infty}\left(1 + \frac{\left(\omega_{LO,i}\right)^2 - \left(\omega_{TO,i}\right)^2}{\left(\omega_{TO,i}\right)^2 - \omega^2 - i\omega\Gamma_i}\right), i = \perp\ or\ \parallel \tag{S6}$$

To get waveguided dispersion relation, we implanted three layers (air-h-BN-Si) models(*39*),

$$q_p = -\frac{\Psi}{d}\left(\tan^{-1}\frac{\varepsilon_1}{\Psi\varepsilon_\perp}\right) + \tan^{-1}\frac{\varepsilon_2}{\Psi\varepsilon_\perp} + m\pi\,, \Psi = -\frac{\sqrt{\varepsilon_\parallel}}{i\sqrt{\varepsilon_\perp}} \tag{S7}$$

Where $q_p = 2\pi k_p$, ε_1 and ε_2 are dielectric functions of top and bottom layers.

For the case of a nano-ray effects, we calculated waveguided dispersion as(*33*),

$$k_p = \frac{2\pi}{\delta(\omega)}\left[l + \alpha(\omega)\right] \tag{S8}$$

Where $\delta(\omega) = 2d\sqrt{-\frac{\varepsilon_\perp}{\varepsilon_\parallel}}$, l is integer index, and $\alpha{\sim}1$ is phase shift acquired at the total internal reflection from a slab surface.

Furthermore, For the colormap in **Fig. S4**, we extract imaginary part of Fresnel reflection coefficient $\Im(r_p)$ calculated by the transfer matrix method(*40*).

Supplementary Note 4. Near-field Simulations

To theoretically corroborate the near-field images observed via PiFM, we performed three-dimensional finite-difference time-domain (FDTD) simulations using Ansys Lumerical FDTD to calculate the electric field intensity distribution $|E|^2$.

The simulated model precisely replicates our experimental device, which comprises a 42-nm-thick hexagonal boron nitride (h-BN) layer integrated onto a silicon photonic crystal (PhC). The PhC consists of equilateral triangular Si pillars, 190 nm in height, with C_{6v} rotational symmetry. This structure is situated on a 2.2-μm-thick SiO_2 layer.

The geometric parameters were defined to match the fabricated devices. The hexagonal lattice with a lattice constant **a** of 4.4 μm, and the side length of each triangular pillar is 1.364 μm. The pillar positions are defined by the distance from the center of the hexagonal unit cell to the nearest vertex of the triangular pillar, which was set to 0.533 μm for the shrunken lattice and 0.795 μm for the expanded lattice. For the simulation, constant refractive indices of $n_{Si} = 3.41$ and $n_{SiO_2} = 1.44$ were used.

To capture the polariton fringe patterns formed in the h-BN layer, the simulation domain was constructed as a hexagonal supercell composed of nine–unit cells. The structure was excited by a normally incident, circularly polarized plane wave at the wavelength corresponding to the RS1 (796 ~ 895 cm^{-1}) resonance. Perfectly Matched Layer (PML) boundary conditions were applied to all boundaries of the simulation domain to absorb outgoing radiation. A two-dimensional frequency-domain monitor was placed within the h-BN layer to map the $|E|^2$ distribution.

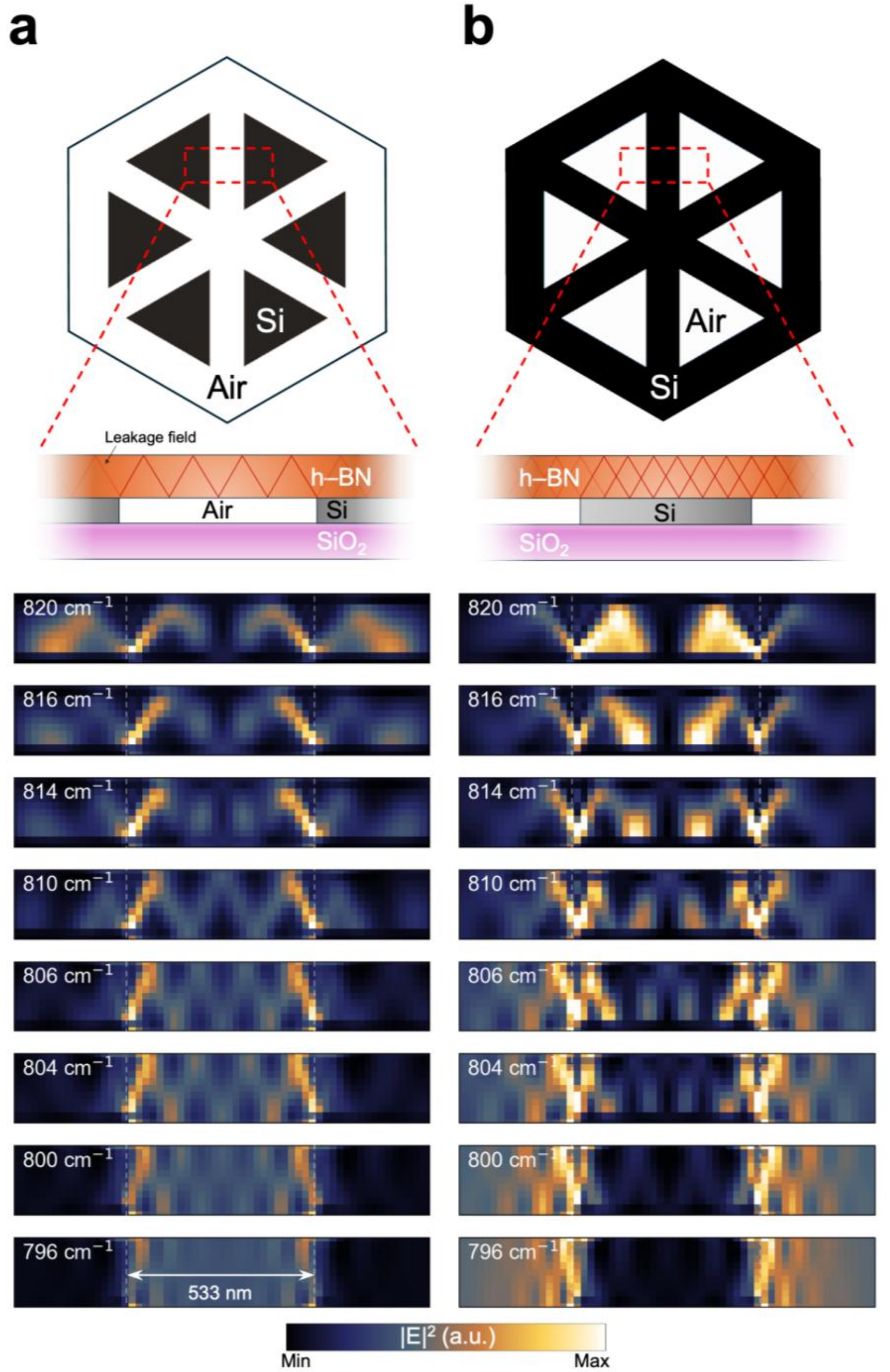

Fig. S6. Cross-sectional simulation from FDTD simulations. (a) Cross-sectional simulation of the cavity structures. (b) Cross-sectional simulation of the inversed geometries structures. The graphics were created by the authors using Microsoft PowerPoint.

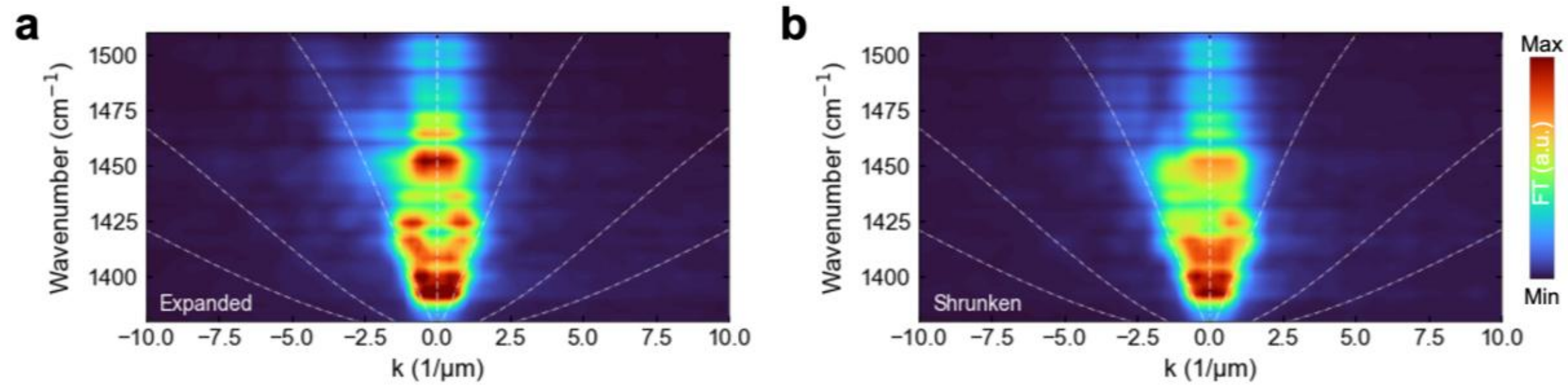

Fig. S7. Dispersion of HPCCs in the RS2 band. (a, b) k_x and k_y dispersion for expanded and shrunken HPCCs, respectively. The white dashed curves represent the dispersion of Si-supported HPhP in the RS2 band.

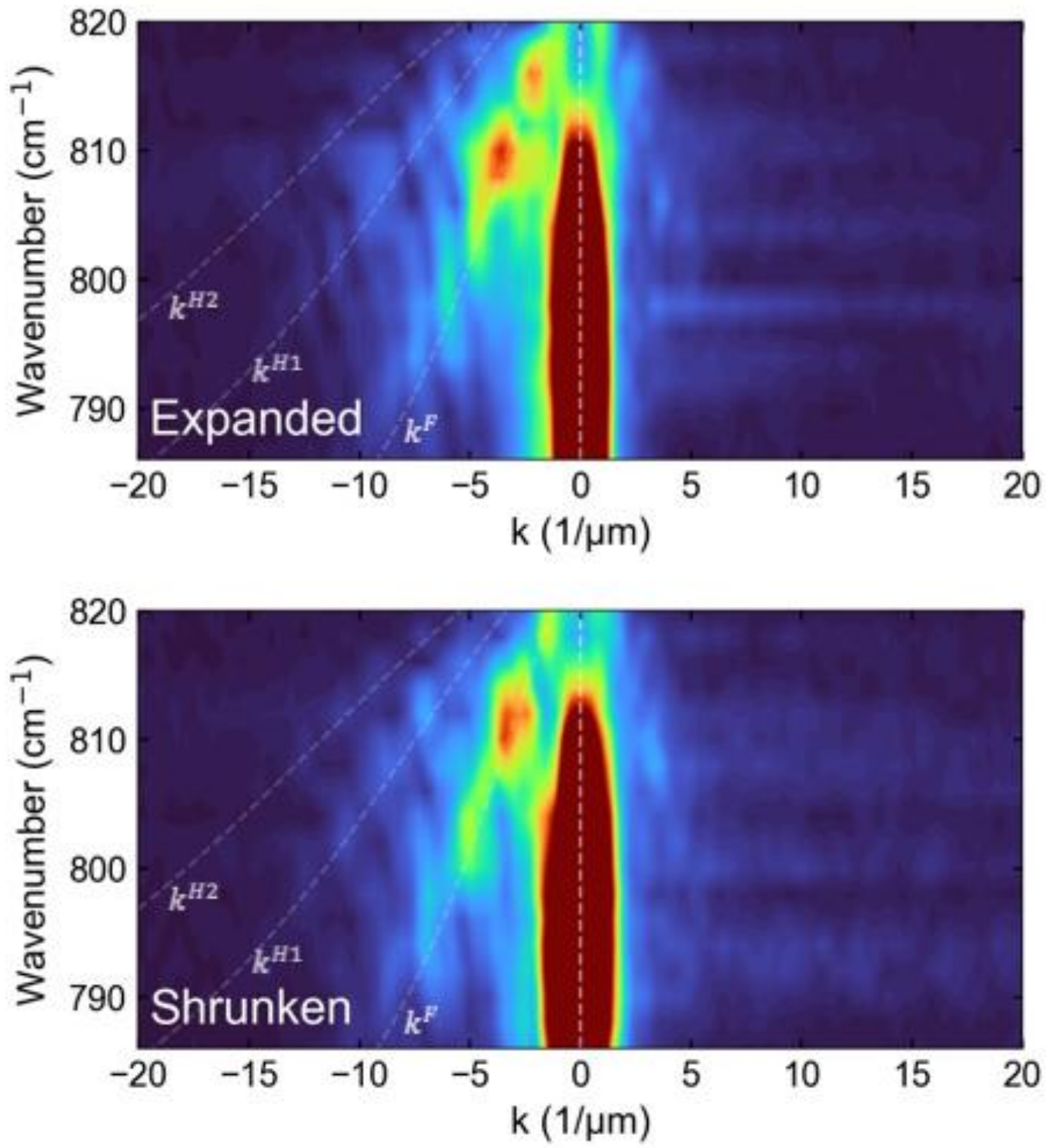

Fig. S8. Dispersion of HPCCs in RS1 band for extended *k*–axis. The white dashed curves represent the calculated dispersions of each mode.

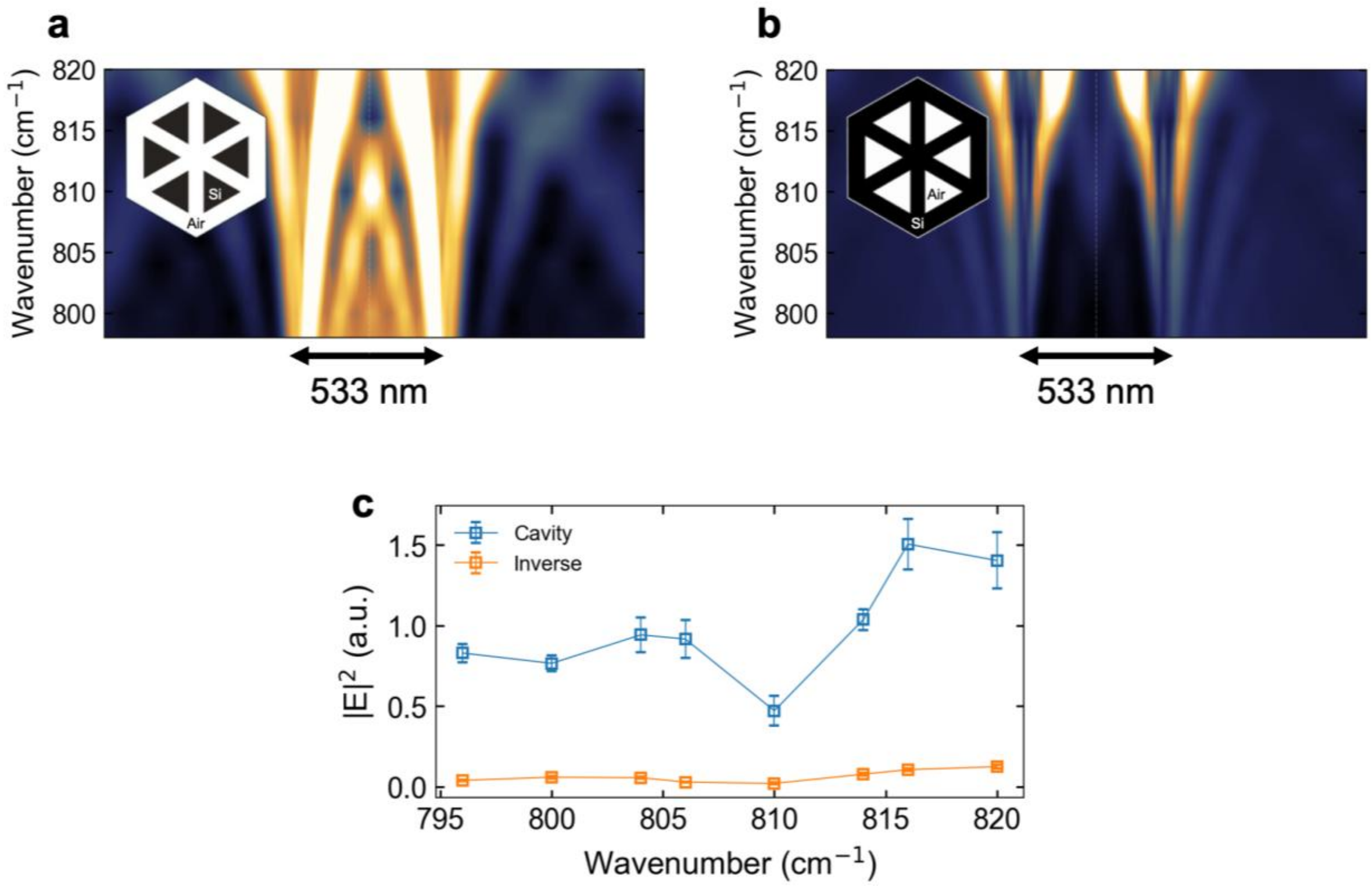

Fig. S9. Line spectra from simulated HPCC and inversed structure. (a) Line spectra of the cavity structures. (b) Line spectra of the inversed structure. Both line spectra were measured at interface between h-BN and air. (c) E-field spectra at center of each structure. The E-field collected at the center of (a) and (b) with 4 pixels linewidth.

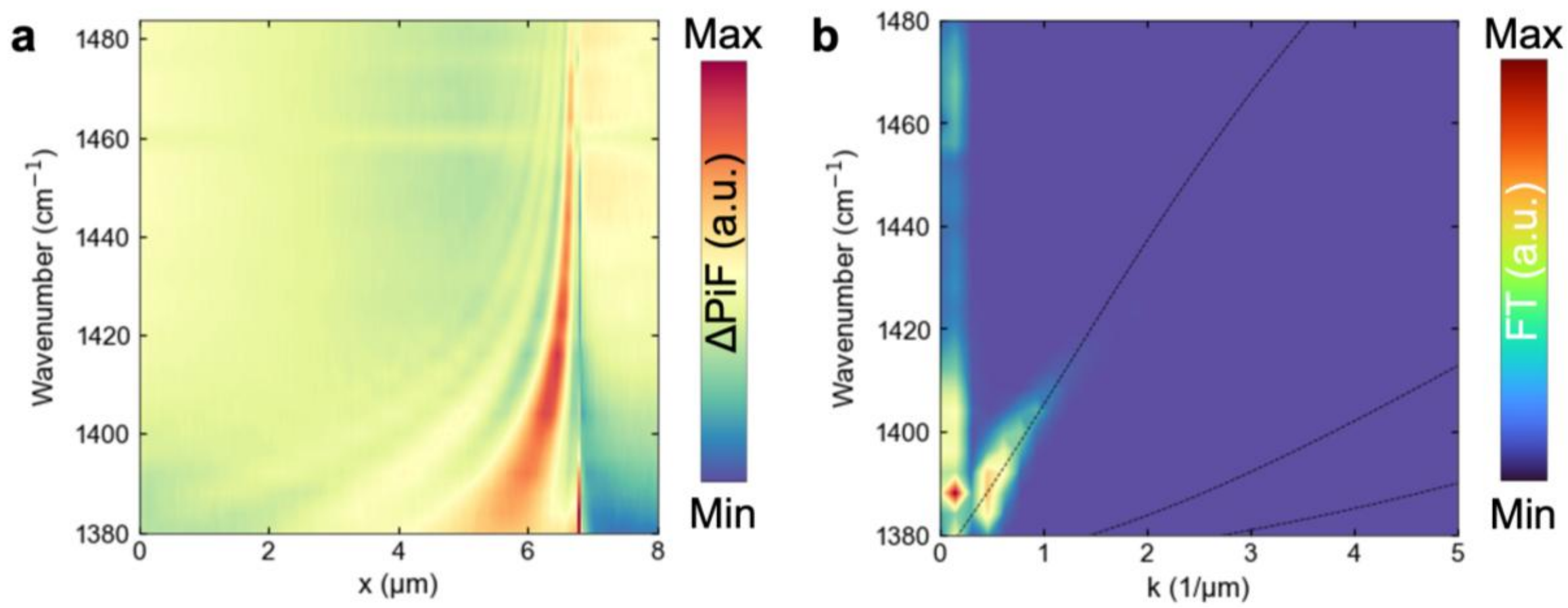

Fig. S10. Dispersion of HPhPs of h-BN on the planar Si substrate in RS2 band. (a) Spectral line profiles of HPhP of h-BN on the planar Si. (b) HPhP dispersion. The dotted line represents calculated dispersion by **Eq. S7**.

Supplementary Note 5. Calculation of Purcell factor

To define the mode volume in the presence of hyperbolic and generally anisotropic dispersive media, we adopt the quasinormal-mode (QNM) formalism(*41*). This approach provides a rigorous and self-consistent definition of the mode volume V_m and the Purcell factor F, which remains valid for open, lossy, and dispersive photonic systems, where the conventional energy-based definition of the mode volume breaks down. Within this framework, the mode volume associated with a given QNM is defined as:

$$V_m = \frac{\int d^3r \left[\tilde{E} \cdot \frac{\partial(\omega\varepsilon)}{\partial\omega} \tilde{E}\right]}{2\varepsilon_0 n^2 |E_{max} \cdot u|^2} \tag{S9}$$

Where, ε is the frequency dependent permittivity tensors, and u is the unit vector defining the dipole orientation. The corresponding Purcell factor is then given by:

$$F = \frac{3}{4\pi^2}\left(\frac{\lambda_0}{n}\right)^3 \frac{Q}{V_m} \tag{S10}$$

where λ_0 is the free-space wavelength, n is the refractive index of the medium, and Q is the quality factor of the resonance. To evaluate the Purcell factor, we extract the spatial field distribution $|E|^2$ from full-wave electromagnetic simulations in the xz-plane cross section (**Fig. S6**). The use of $|E|^2$ in defining the mode volume is justified by the predominantly TM-like character of the polaritonic mode, for which the electric field is dominated by the out-of-plane component E_z. Under this assumption, the electromagnetic energy density is mainly governed by the ε_z component of the permittivity tensor. Consequently, contributions from in-plane field components are neglected, and the mode volume is evaluated using the simulated $|E_z|^2$ (approximated here by $|E|^2$) distribution weighted by ε_z. The quality factor Q is obtained experimentally from the measured linewidth of the resonance peak (**Fig. 5c**), yielding Q ~281 for the shrunken structure. At the frequency of 816 cm^{-1}, both simulations and experiments consistently reveal an antinode of the electric field at the center of the cavity. This agreement validates the use of the simulated QNM field at this frequency for the mode-volume evaluation. Using **Eq. S9**, we obtain a normalized mode volume of $\bar{V}_m = 4.3 \times 10^{-7}\ (\lambda_0/n)^3$ which leads to a Purcell factor $F = 4.93 \times 10^7$ via **Eq. S10**. This exceptionally large Purcell enhancement originates from the combination of extreme field confinement and high-quality factor in the dielectric photonic structure, highlighting its potential for strongly enhanced light–matter interactions mediated by polaritonic quantized modes.

Supplementary Note 6. Role of the photonic-crystal

To clarify the role of the PhC lattice, we performed FDTD-calculated local density of states (LDOS) simulations for h-BN on the PhC structure and on planar Si. The h-BN/PhC structure exhibits a higher LDOS over the RS1 spectral range than h-BN/planar Si, indicating that the PhC environment increases the local polaritonic mode density. Since the local energy-dissipation rate of a dipole is proportional to the LDOS, this enhancement is consistent with stronger cavity-field confinement and an enhanced Purcell response. These results show that the PhC geometry modifies the RS1 polaritonic environment relative to planar Si and provides additional local optical states that support the observed cavity response.

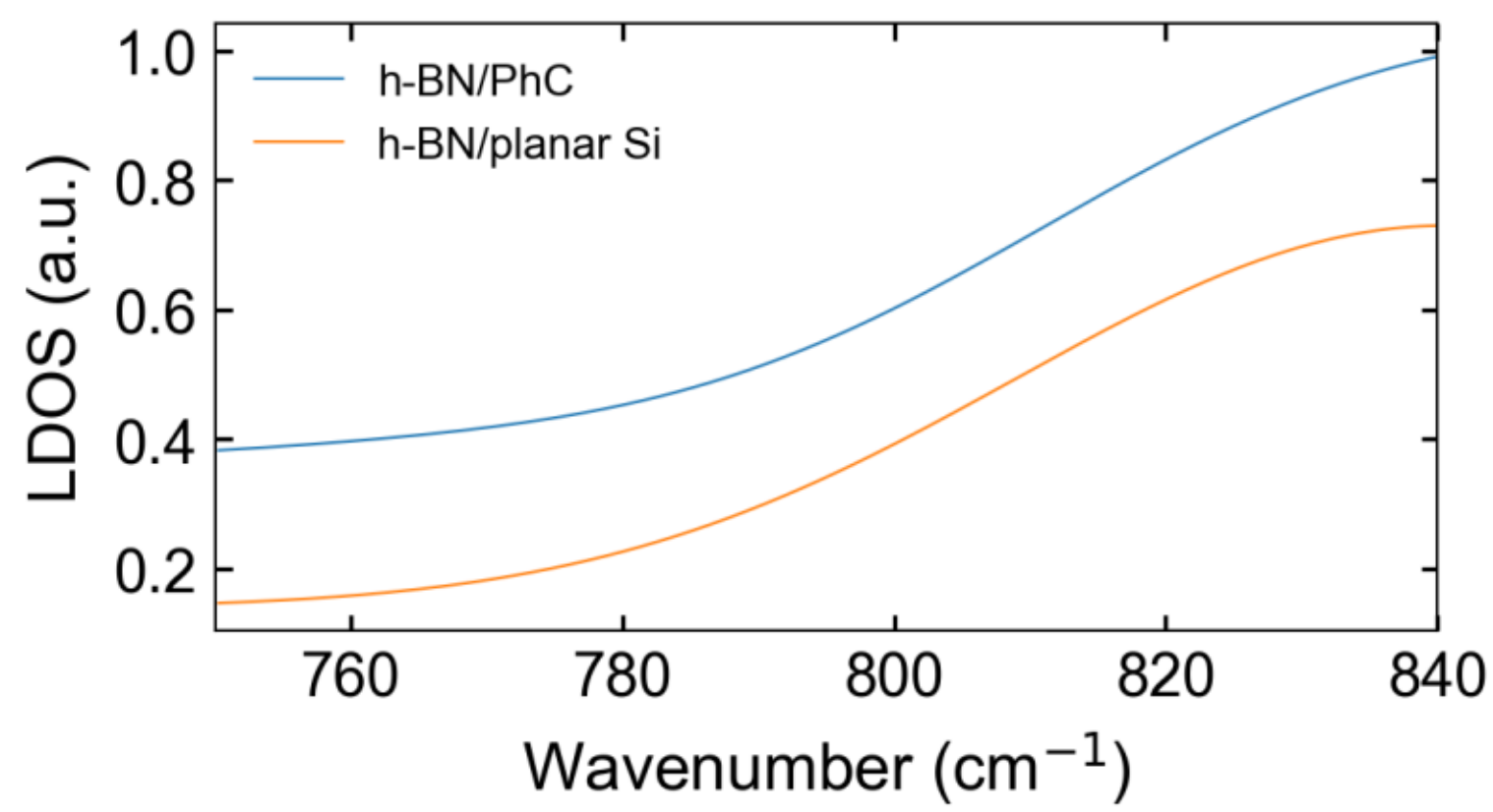

Fig. S11. FDTD-calculated LDOS spectra for h-BN on the PhC structure and h-BN on planar Si. The h-BN/PhC structure shows an enhanced LDOS over the RS1 spectral range, indicating that the PhC environment increases the local polaritonic mode density and supports stronger cavity-field confinement.

REFERENCES

1. J. D. Caldwell, L. Lindsay, V. Giannini, I. Vurgaftman, T. L. Reinecke, S. A. Maier, O. J. Glembocki, Low-loss, infrared and terahertz nanophotonics using surface phonon polaritons. *Nanophotonics* **4**, 44–68 (2014).

2. J. Duan, A. T. Martín-Luengo, C. Lanza, S. Partel, K. Voronin, A. I. F. Tresguerres-Mata, G. Álvarez-Pérez, A. Y. Nikitin, J. Martín-Sánchez, P. Alonso-González, Canalization-based super-resolution imaging using an individual van der Waals thin layer. *Sci. Adv.* **11**, eads0569 (2025).

3. K. Wang, Z. Huang, L. Xiong, K. Wang, Y. Bai, H. Long, N. Deng, B. Wang, G. Hu, P. Lu, Observation of canalized phonon polaritons in a single α-MoO_3 flake. *Optica* **12**, 343–349 (2025).

4. A. I. F. Tresguerres-Mata, C. Lanza, J. Taboada-Gutiérrez, J. R. Matson, G. Álvarez-Pérez, M. Isobe, A. Tarazaga Martín-Luengo, J. Duan, S. Partel, M. Vélez, J. Martín-Sánchez, A. Y. Nikitin, J. D. Caldwell, P. Alonso-González, Observation of naturally canalized phonon polaritons in LiV_2O_5 thin layers. *Nat. Commun.* **15**, 2696 (2024).

5. X. Lin, Y. Yang, N. Rivera, J. J. López, Y. Shen, I. Kaminer, H. Chen, B. Zhang, J. D. Joannopoulos, M. Soljačić, All-angle negative refraction of highly squeezed plasmon and phonon polaritons in graphene–boron nitride heterostructures. *Proc. Natl. Acad. Sci. U.S.A.* **114**, 6717–6721 (2017).

6. H. Hu, N. Chen, H. Teng, R. Yu, M. Xue, K. Chen, Y. Xiao, Y. Qu, D. Hu, J. Chen, Z. Sun, P. Li, F. J. G. de Abajo, Q. Dai, Gate-tunable negative refraction of mid-infrared polaritons. *Science* **379**, 558–561 (2023).

7. H. Herzig Sheinfux, L. Orsini, M. Jung, I. Torre, M. Ceccanti, S. Marconi, R. Maniyara, D. Barcons Ruiz, A. Hötger, R. Bertini, S. Castilla, N. C. H. Hesp, E. Janzen, A. Holleitner, V. Pruneri, J. H. Edgar, G. Shvets, F. H. L. Koppens, High-quality nanocavities through multimodal confinement of hyperbolic polaritons in hexagonal boron nitride. *Nat. Mater.* **23**, 499–505 (2024).

8. X. Guo, N. Li, X. Yang, R. Qi, C. Wu, R. Shi, Y. Li, Y. Huang, F. J. García de Abajo, E.-G. Wang, P. Gao, Q. Dai, Hyperbolic whispering-gallery phonon polaritons in boron nitride nanotubes. *Nat. Nanotechnol.* **18**, 529–534 (2023).

9. G. Ramer, M. Tuteja, J. R. Matson, M. Davanco, T. G. Folland, A. Kretinin, T. Taniguchi, K. Watanabe, K. S. Novoselov, J. D. Caldwell, A. Centrone, High-Q dark hyperbolic phonon-polaritons in hexagonal boron nitride nanostructures. *Nanophotonics* **9**, 1457–1467 (2020).

10. L. Zhou, X. Ni, Z. Wang, E. M. Renzi, J. Xu, Z. Zhou, Y. Yin, Y. Yin, R. Song, Z. Zhao, K. Yu, D. Huang, Z. Wang, X. Cheng, A. Alù, T. Jiang, Engineering shear polaritons in 2D twisted heterostructures. *Nat. Commun.* **16**, 2953 (2025).

11. Z. Zhou, R. Song, J. Xu, X. Ni, Z. Dang, Z. Zhao, J. Quan, S. Dong, W. Hu, D. Huang, K. Chen, Z. Wang, X. Cheng, M. B. Raschke, A. Alù, T. Jiang, Gate-tuning hybrid polaritons in twisted α-MoO_3/graphene heterostructures. *Nano Lett.* **23**, 11252–11259 (2023).

12. M. Chen, X. Lin, T. H. Dinh, Z. Zheng, J. Shen, Q. Ma, H. Chen, P. Jarillo-Herrero, S. Dai, Configurable phonon polaritons in twisted α-MoO_3. *Nat. Mater.* **19**, 1307–1311 (2020).

13. L. Dou, J. Xu, Z. Zhou, S. Li, Z. Wei, T. Jiang, Z. Wang, X. Cheng, Two-dimensional negative refraction steering of hybrid polaritons in graphene/twisted bilayer α-MoO_3 heterostructures. *Opt. Express* **33**, 3881–3889 (2025).

14. J. Álvarez-Cuervo, M. Obst, S. Dixit, G. Carini, A. I. F. Tresguerres-Mata, C. Lanza, E. Terán-García, G. Álvarez-Pérez, L. F. Álvarez-Tomillo, K. Diaz-Granados, R. Kowalski, A. S. Senerath, N. S. Mueller, L. Herrer, J. M. De Teresa, S. Wasserroth, J. M. Klopf, T. Beechem, M. Wolf, L. M. Eng, T. G. Folland, A. Tarazaga Martín-Luengo, J. Martín-Sánchez, S. C. Kehr, A. Y. Nikitin, J. D. Caldwell, P. Alonso-González, A. Paarmann, Unidirectional ray polaritons in twisted asymmetric stacks. *Nat. Commun.* **15**, 9042 (2024).

15. A. I. F. Tresguerres-Mata, O. G. Matveeva, C. Lanza, J. Álvarez-Cuervo, K. V. Voronin, F. Calavalle, G. Avedissian, P. Díaz-Núñez, G. Álvarez-Pérez, A. Tarazaga Martín-Luengo, J. Taboada-Gutiérrez, J. Duan, J. Martín-Sánchez, A. Bylinkin, R. Hillenbrand, A. Mishchenko, L.

E. Hueso, V. S. Volkov, A. Y. Nikitin, P. Alonso-González, Directional strong coupling at the nanoscale between hyperbolic polaritons and organic molecules. *Nat. Photonics* **19**, 1196–1202 (2025).

16. W. Lyu, H. Teng, C. Wu, X. Zhang, X. Guo, X. Yang, Q. Dai, Anisotropic acoustic phonon polariton-enhanced infrared spectroscopy for single molecule detection. *Nanoscale* **13**, 12720–12726 (2021).

17. P. Li, I. Dolado, F. J. Alfaro-Mozaz, F. Casanova, L. E. Hueso, S. Liu, J. H. Edgar, A. Y. Nikitin, S. Vélez, R. Hillenbrand, Infrared hyperbolic metasurface based on nanostructured van der Waals materials. *Science* **359**, 892–896 (2018).

18. J. D. Caldwell, A. V. Kretinin, Y. Chen, V. Giannini, M. M. Fogler, Y. Francescato, C. T. Ellis, J. G. Tischler, C. R. Woods, A. J. Giles, M. Hong, K. Watanabe, T. Taniguchi, S. A. Maier, K. S. Novoselov, Sub-diffractional volume-confined polaritons in the natural hyperbolic material hexagonal boron nitride. *Nat. Commun.* **5**, 5221 (2014).

19. J. Lv, Y. Wu, J. Liu, Y. Gong, G. Si, G. Hu, Q. Zhang, Y. Zhang, J.-X. Tang, M. S. Fuhrer, H. Chen, S. A. Maier, C.-W. Qiu, Q. Ou, Hyperbolic polaritonic crystals with configurable low-symmetry Bloch modes. *Nat. Commun.* **14**, 3894 (2023).

20. J. Yang, Z. E. Krix, S. Kim, J. Tang, M. Mayyas, Y. Wang, K. Watanabe, T. Taniguchi, L. H. Li, A. R. Hamilton, I. Aharonovich, O. P. Sushkov, K. Kalantar-Zadeh, Near-field excited archimedean-like tiling patterns in phonon-polaritonic crystals. *ACS Nano* **15**, 9134–9142 (2021).

21. A. Fali, S. T. White, T. G. Folland, M. He, N. A. Aghamiri, S. Liu, J. H. Edgar, J. D. Caldwell, R. F. Haglund, Y. Abate, Refractive index-based control of hyperbolic phonon-polariton propagation. *Nano Lett.* **19**, 7725–7734 (2019).

22. S. G. Menabde, Y. Lim, K. Voronin, J. T. Heiden, A. Y. Nikitin, S. Lee, M. S. Jang, Polaritonic Fourier crystal. *Nat. Commun.* **16**, 2530 (2025).

23. Y. Chen, Y. Francescato, J. D. Caldwell, V. Giannini, T. W. W. Maß, O. J. Glembocki, F. J. Bezares, T. Taubner, R. Kasica, M. Hong, S. A. Maier, Spectral tuning of localized surface phonon polariton resonators for low-loss mid-IR applications. *ACS Photonics* **1**, 718–724 (2014).

24. H. Wang, J. Li, J. H. Edgar, X. G. Xu, Three-dimensional near-field analysis through peak force scattering-type near-field optical microscopy. *Nanoscale* **12**, 1817–1825 (2020).

25. K. Srinivasan, O. Painter, Momentum space design of high-Q photonic crystal optical cavities. *Opt. Express* **10**, 670–684 (2002).

26. R. Chen, Y. Zheng, X. Huang, Q. Lin, C. Ye, M. Xiong, M. Wubs, Y. Ma, M. Pu, S. Xiao, Observation of multiple bulk bound states in the continuum modes in a photonic crystal cavity. *Beilstein J. Nanotechnol.* **14**, 544–551 (2023).

27. Y. Akahane, T. Asano, B.-S. Song, S. Noda, Fine-tuned high-Q photonic-crystal nanocavity. *Opt. Express* **13**, 1202–1214 (2005).

28. Z. Shi, H. A. Bechtel, S. Berweger, Y. Sun, B. Zeng, C. Jin, H. Chang, M. C. Martin, M. B. Raschke, F. Wang, Amplitude- and phase-resolved nanospectral imaging of phonon polaritons in hexagonal boron nitride. *ACS Photonics* **2**, 790–796 (2015).

29. A. Ambrosio, M. Tamagnone, K. Chaudhary, L. A. Jauregui, P. Kim, W. L. Wilson, F. Capasso, Selective excitation and imaging of ultraslow phonon polaritons in thin hexagonal boron nitride crystals. *Light Sci. Appl.* **7**, 27 (2018).

30. E. Yoxall, M. Schnell, A. Y. Nikitin, O. Txoperena, A. Woessner, M. B. Lundeberg, F. Casanova, L. E. Hueso, F. H. L. Koppens, R. Hillenbrand, Direct observation of ultraslow hyperbolic polariton propagation with negative phase velocity. *Nat. Photonics* **9**, 674–678 (2015).

31. J. Chen, M. Badioli, P. Alonso-González, S. Thongrattanasiri, F. Huth, J. Osmond, M. Spasenović, A. Centeno, A. Pesquera, P. Godignon, A. Zurutuza Elorza, N. Camara, F. J. G. de Abajo, R. Hillenbrand, F. H. L. Koppens, Optical nano-imaging of gate-tunable graphene plasmons. *Nature* **487**, 77–81 (2012).

32. J. Jahng, E. O. Potma, E. S. Lee, Nanoscale spectroscopic origins of photoinduced tip–sample force in the midinfrared. *Proc. Natl. Acad. Sci. U.S.A.* **116**, 26359–26366 (2019).

33. S. Dai, Q. Ma, T. Andersen, A. S. Mcleod, Z. Fei, M. K. Liu, M. Wagner, K. Watanabe, T. Taniguchi, M. Thiemens, F. Keilmann, P. Jarillo-Herrero, M. M. Fogler, D. N. Basov, Subdiffractional focusing and guiding of polaritonic rays in a natural hyperbolic material. *Nat. Commun.* **6**, 6963 (2015).

34. P. Li, M. Lewin, A. V. Kretinin, J. D. Caldwell, K. S. Novoselov, T. Taniguchi, K. Watanabe, F. Gaussmann, T. Taubner, Hyperbolic phonon-polaritons in boron nitride for near-field optical imaging and focusing. *Nat. Commun.* **6**, 7507 (2015).

35. N. Chen, H. Teng, H. Hu, F. J. García de Abajo, R. Hillenbrand, Q. Dai, Boundary-induced excitation of higher-order hyperbolic phonon polaritons. *Nat. Photonics* **19**, 1225–1232 (2025).

36. M. Jang, S. G. Menabde, F. Kiani, J. T. Heiden, V. A. Zenin, N. Asger Mortensen, G. Tagliabue, M. S. Jang, Fourier analysis of near-field patterns generated by propagating polaritons. *Phys. Rev. Appl.* **22**, 014076 (2024).

37. J. Jahng, B. Kim, E. S. Lee, Quantitative analysis of photoinduced thermal force: Surface and volume responses. *Phys. Rev. B* **106**, 155424 (2022).

38. A. Anjum Sifat, J. Jahng, E. O. Potma, Photo-induced force microscopy (PiFM) – principles and implementations. *Chem. Soc. Rev.* **51**, 4208–4222 (2022).

39. S. Dai, Z. Fei, Q. Ma, A. S. Rodin, M. Wagner, A. S. McLeod, M. K. Liu, W. Gannett, W. Regan, K. Watanabe, T. Taniguchi, M. Thiemens, G. Dominguez, A. H. C. Neto, A. Zettl, F. Keilmann, P. Jarillo-Herrero, M. M. Fogler, D. N. Basov, Tunable phonon polaritons in atomically thin van der Waals crystals of boron nitride. *Science* **343**, 1125–1129 (2014).

40. N. C. Passler, A. Paarmann, Generalized 4 × 4 matrix formalism for light propagation in anisotropic stratified media: Study of surface phonon polaritons in polar dielectric heterostructures. *J. Opt. Soc. Am. B* **34**, 2128–2139 (2017).

41. C. Sauvan, J. P. Hugonin, I. S. Maksymov, P. Lalanne, Theory of the spontaneous optical emission of nanosize photonic and plasmon resonators. *Phys. Rev. Lett.* **110**, 237401 (2013).
